# Sphingolipids in neurodegenerative diseases

**DOI:** 10.3389/fnins.2023.1137893

**Published:** 2023-02-16

**Authors:** Xueyang Pan, Debdeep Dutta, Shenzhao Lu, Hugo J. Bellen

**Affiliations:** ^1^Department of Molecular and Human Genetics, Baylor College of Medicine, Houston, TX, United States; ^2^Jan and Dan Duncan Neurological Research Institute, Texas Children’s Hospital, Houston, TX, United States; ^3^Department of Neuroscience, Baylor College of Medicine, Houston, TX, United States

**Keywords:** *Drosophila*, neurodegeneration, sphingolipids, ceramides, mitochondria, lysosome, Parkinson’s disease

## Abstract

Neurodegenerative Diseases (NDDs) are a group of disorders that cause progressive deficits of neuronal function. Recent evidence argues that sphingolipid metabolism is affected in a surprisingly broad set of NDDs. These include some lysosomal storage diseases (LSDs), hereditary sensory and autonomous neuropathy (HSAN), hereditary spastic paraplegia (HSP), infantile neuroaxonal dystrophy (INAD), Friedreich’s ataxia (FRDA), as well as some forms of amyotrophic lateral sclerosis (ALS) and Parkinson’s disease (PD). Many of these diseases have been modeled in *Drosophila* melanogaster and are associated with elevated levels of ceramides. Similar changes have also been reported in vertebrate cells and mouse models. Here, we summarize studies using fly models and/or patient samples which demonstrate the nature of the defects in sphingolipid metabolism, the organelles that are implicated, the cell types that are initially affected, and potential therapeutics for these diseases.

## Introduction

Neurodegenerative diseases (NDDs) are a group of progressive disorders that cause physiological deficits in the nervous system. Depending on the nature of the NDD, specific brain areas and neuronal subtypes are affected first and other neuronal subtypes often become affected during disease progression. Symptoms, such as decline in motor and cognitive functions, often also worsen with time. However, for many NDDs the initial pathological changes are insidious and observable symptoms do not arise until the disease is at a more advanced stage. For example, in Parkinson’s disease (PD), a considerable loss of dopaminergic neurons occurs before canonical PD-associated symptoms are evident (Fearnley and Lees, 1991; Greffard et al., 2006). An early diagnosis of NDDs requires an in-depth understanding of the underlying mechanisms as well as the identification of useful biomarkers that are detectable during the latent period of the diseases.

Most of our knowledge of the pathogenesis of NDDs are derived from studying the function of genes that are associated with these diseases. Many NDDs with low prevalence have a monogenic etiology. Even for more common NDDs, such as PD and amyotrophic lateral sclerosis (ALS), where the majority of cases are sporadic, the understanding of the disease mechanism heavily relies on studies of familial cases and disease-causing genes/variants. Interestingly, some rare NDD genes are also identified as risk factors for common NDDs, suggesting that the pathogenesis of different NDDs share similar molecular mechanisms (Ma et al., 2022). This is corroborated by similar pathological defects observed in various NDDs, including the presence of aberrant protein deposits and aggregates as well as deficits in lysosomal and mitochondrial functions (Soto and Pritzkow, 2018; Wallings et al., 2019; Monzio Compagnoni et al., 2020; Nixon, 2020). Recent studies indicate that sphingolipids (SLs) are key players in a surprisingly broad set of NDDs. The importance of SLs in NDDs is supported by the association of genes that play a role in SL metabolism and the changes in SL profile in patients and disease models.

Fruit flies are a premier model organism for genetic studies (Bellen et al., 2010). The conservation between fly and human genes and the sophisticated nervous system of flies allows for the study of NDDs in flies (Bellen et al., 2019; Ma et al., 2022). A forward genetic screen on the *Drosophila* X chromosome identified 165 genes that are required for the development, function, and maintenance of the nervous system (Yamamoto et al., 2014). More than 90% of these genes are conserved in humans and currently, more than 65% of the identified genes have been associated with diseases based on the Online Mendelian Inheritance in Man (OMIM) (Amberger et al., 2019). Recently powerful genetic tools have been developed in flies that allow for in-depth analysis of disease-associated genes. This includes the T2A-GAL4 gene trap technology which permits the determination of gene expression patterns and protein subcellular localization, assessment of loss-of-function phenotypes, humanization of the fly models, assessment of impacts of human variants on gene function, tissue-specific and reversible removal of proteins, and identification of interacting proteins *in vivo* (Diao et al., 2015; Nagarkar-Jaiswal et al., 2015; Lee et al., 2018; Li-Kroeger et al., 2018; Bellen et al., 2019; Kanca et al., 2019, 2022).

In this review, we will focus on the NDDs that are associated with changes in SL metabolism. We will first summarize the SL metabolic pathway. We then introduce SL-related NDDs and the models generated in flies. We summarize the symptoms observed in patients and phenotypes associated with disease models with a focus on changes in SL metabolism. We explore how elevated SLs are a common pathogenic factor during the progression of NDDs and discuss future issues.

## Overview of sphingolipid metabolism

**FIGURE 1:**
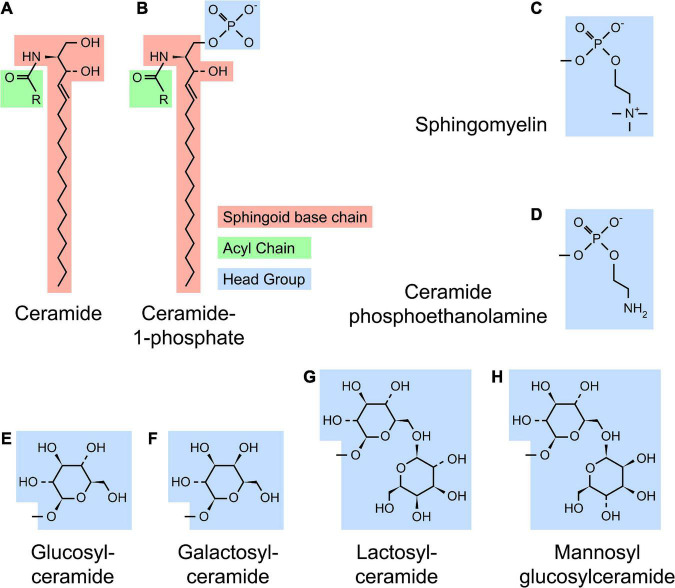
Structure of sphingolipids. **(A)** A typical SL [such as ceramide (Cer)] contains a sphingoid base (red) and a N-linked acyl chain (green). **(B)** Complex SLs have an O-linked head group (blue) on the ceramide backbone [ceramide-1-phosphate (C1P) with a phosphate headgroup as shown]. **(C–H)** Several examples of SL headgroups observed in human and flies are shown. Humans produce all SL species except mannosyl glucosylceramide (MacCer), while flies produce ceramide phosphoethanolamine (CPE), glucosylceramide (GlcCer) and MacCer. All higher order glycosphingolipids (GSLs) are synthesized from lactosylceramide (LacCer) in human or MacCer in flies.

**FIGURE 2:**
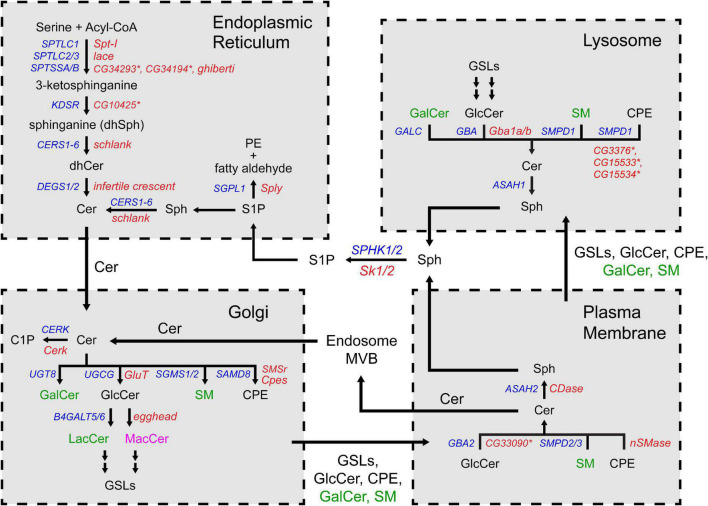
The sphingolipid metabolism network. The biochemical reactions are grouped by the organelles in which they occur. The substrates and products are indicated in three colors depending on their presence in human and flies: black (both human and fly), green (human) and magenta (fly). The genes encoding the enzymes are also indicated in colors: blue (human) and red (fly). Asterisks indicate predicted orthologs in flies. Cer, ceramide; dhCer, dihydroceramide; C1P, ceramide-1-phosphate; GalCer, galactosylceramide; GlcCer, glucosylceramide; LacCer, lactosylceramide; MacCer, mannosyl glucosylceramide; PE, phosphoethanolamine; CPE, ceramide phosphoethanolamine; SM, sphingomyelin; Sph, sphingosine; dhSph, dihydrosphingosine; S1P, sphingosine-1-phosphate; GSL, glycosphingolipid.

Sphingolipid refers to a class of lipids that contain a sphingoid base, a long chain amino alcohol moiety (red in Figure 1). The majority of SLs also have an N-linked acyl chain (green in Figure 1), and a hydrophilic head group (blue in Figure 1). The metabolism of SLs involves a complex biochemical network that occurs in different cellular compartments (Figure 2). Each pathway is tightly controlled by metabolic enzymes (Figure 2 and Table 1). Here we briefly introduce these pathways, the genes and enzymes involved in the pathways, and compare SL metabolism in human and flies.

**TABLE 1:** List of human SL metabolism genes and the fly orthologs.

Human genes	Fly genes	DIOPT score^†^ (out of 16)	Activity	References^††^
**Sphingolipid *de novo* synthesis pathway**				
*SPTLC1*	*Spt-I*	15	Serine palmitoyltransferase, serine + acyl-CoA → 3-ketosphinganine	Oswald et al., 2015
*SPTLC2/3*	*lace*	15 (*SPTLC2*)		Adachi-Yamada et al., 1999
*SPTSSA/B*	CG34293*/ CG34194*/ghi	8 (*SPTSSA-CG34293*)		Guan et al., 2013
*ORMDL1/2/3*	*Ormdl*	14 (*ORMDL3*)	SPT activity suppression	Hjelmqvist et al., 2002
*KDSR*	CG10425*	15	3-keto-dhSph reductase, 3-ketosphinganine → dhSph	
*CERS 1/2/3/4/5/6*	*schlank*	13 (*CERS5/6*)	Ceramide synthase, dhSph + acyl-CoA → dhCer Sph + acyl-CoA → Cer	Bauer et al., 2009
*DEGS1/2*	*ifc*	15 (*DEGS2*)	SL delta(4)-desaturase, dhCer → Cer	Ternes et al., 2002
**SM and CPE synthesis**				
*CERT1*	*Cert*	14	ER–Golgi Cer trafficking	Rao et al., 2007
*SGMS1/2*	No fly ortholog	N/A	SM synthase, Cer + phosphatidylcholine → SM + DG	
*SAMD8*	*SMSr*	13	CPE synthase, Cer + phosphatidylethanolamine → CPE + DG	Vacaru et al., 2013
No human ortholog	*Cpes*	N/A	CPE synthase, Cer + CDP-ethanolamine → CPE + CMP	
**GSL synthesis (mono- and di- glycosyl ceramide)**				
*UGCG*	*GlcT*	15	Cer glucosyltransferase, Cer + UDP-glucose → GlcCer + UDP	Kohyama-Koganeya et al., 2004
*UGT8*	Ugt50B3*	10	Cer galactosyltransferase, Cer + UDP-galactose → GalCer + UDP	
*B4GALT5/6*	No fly ortholog	N/A	GlcCer beta-1,4-galactosyltransferase, GlcCer + UDP-galactose → LacCer + UDP	
No human ortholog	*egh*	N/A	GlcCer glycosyltransferase, GlcCer + GDP-mannose → MacCer + GDP	Wandall et al., 2003
**SM and salvage pathways**				
*PSAP*	*Sap-r*	12	Facilitate GSL hydrolysis	Hindle et al., 2017; Sellin et al., 2017
*SMPD1*	CG3376*/ CG15533*/ CG15534*	15 (*CG3376*)	Acidic sphingomyelinase, SM → Cer + PC (human)	
*SMPD2*	*nSMase*	15	Neutral sphingomyelinase, SM → Cer + PC (human) CPE → Cer + PE (fly)	Chen et al., 2022
*SMPD3*	No fly ortholog	N/A	Neutral sphingomyelinase, SM → Cer + PC (human)	
*SMPD4*	CG6962*	15		
*SMPD5*	No fly ortholog	N/A		
*ENPP7*	No fly ortholog	N/A	Alkaline sphingomyelinase, SM → Cer + PC (human)	
*GBA*	*Gba1a/b*	14 (*Gba1a/b*)	Acidic glucosylceramidase, GlcCer → Cer + Glucose	Davis et al., 2016
*GBA2*	CG33090*	15	Neutral glucosylceramidase, GlcCer → Cer + Glucose	
*GALC*	No fly ortholog	N/A	Acidic galactosylceramidase, GalCer → Cer + Galactose	
*ASAH1*	No fly ortholog	N/A	Acidic ceramidase, Cer → Sph + fatty acid	
*ASAH2*	*Cdase*	9	Neutral ceramidase, Cer → Sph + fatty acid^#^	Yuan et al., 2011
*ACER1/2/3*	*bwa*	14 (*ACER2*)		
*SGPP1/2*	No fly ortholog	N/A	S1P phosphatase, S1P → Sph	
**S1P synthesis and catabolism**				
*SPHK1/2*	*Sk1/2*	13 (*SPHK1-Sk2*)	Sph kinase, Sph → S1P	Herr et al., 2004
*SGPL1*	*Sply*	15	S1P lyase, S1P → PE + fatty aldehyde	Herr et al., 2003
**C1P synthesis**				
*CERK*	*Cerk*	14	Ceramide kinase, Cer → C1P	Dasgupta et al., 2009

^†^DRSC integrative ortholog prediction tool (DIOPT) score indicates the level of homology between human and fly orthologs (Hu et al., 2011). The score is evaluated for each pair of human-fly orthologs. A high score indicates a high level of homology between orthologs, and the maximum score is 16. Only the highest score is listed in the table, and the gene symbol indicates the ortholog or the human-fly ortholog pairs that receive the score.

^††^The references indicate the studies on the fly orthologs.

*Predicted orthologs in flies.

^#^Fly Bwa does not have ceramidase activity.

SPT, serine palmitoyltransferase; Sph, sphingosine; dhSph, dihydrosphingosine; Cer, ceramide; dhCer, dihydroceramide; SM, sphingomyelin; PC, phosphocholine; CPE, ceramide phosphoethanolamine; PE, phosphoethanolamine; GlcCer, glucosylceramide; GalCer, galactosylceramide; LacCer, lactosylceramide; MacCer, mannosyl glucosylceramide; GSL, glycosphingolipid; S1P, sphingosine-1-phosphate; C1P, ceramide-1-phosphate.

### SL *de novo* synthesis

The *de novo* synthesis of SLs occurs in the endoplasmic reticulum (ER) (Figure 2, top left). The first, and rate limiting, reaction in the pathway is the condensation of L-serine and acyl-CoA to produce 3-keto-dihydrosphingosine. This reaction is catalyzed by serine palmitoyltransferase (SPT), an enzyme complex containing three subunits SPTLC1, SPTLC2/3, and SPTSSA/B. Next, 3-keto-dihydrosphingosine is processed into ceramide, the central molecule in the SL metabolic network, by three sequential reactions. The activity of the pathway is regulated by the ORMDL protein. ORMDL interacts with SPT and suppresses SPT activity in the presence of excessive ceramide, forming a negative feedback loop (Hjelmqvist et al., 2002; Siow and Wattenberg, 2012; Gupta et al., 2015). Flies have orthologs of all the genes that encode the SL *de novo* synthesis enzymes. *Schlank*, the fly gene encoding ceramide synthase, is the only ortholog of six human genes (*CERS1-6*) (Bauer et al., 2009).

### Complex SL synthesis

Ceramide is transported from the ER to the Golgi by a specific transporter CERT (Hanada et al., 2003; Rao et al., 2007). Complex SLs, which refer to the SLs that have an O-linked head group, are synthesized in the Golgi apparatus (Figure 2, bottom left). Complex SLs include glycosphingolipids (GSLs, carbohydrate head groups), sphingomyelin (SM, phosphocholine headgroup) and ceramide phosphoethanolamine [CPE, phosphoethanolamine (PE) headgroup]. Unlike SM and CPE which have unique headgroup, GSLs are very diverse in their headgroups. The simplest GSLs are glucosylceramide (GlcCer) and galactosylceramide (GalCer), which have a single sugar headgroup. All higher order GSLs (GSLs with extended sugar chain) are derived from GlcCer by adding a galactose first (lactosylceramide, LacCer) followed by branched saccharide chains of different length and sugar composition (D’Angelo et al., 2013). Upon their synthesis, the complex SLs are transferred to the plasma membrane through vesicular transport.

Flies have an ortholog of ceramide glucosyltransferase (*GlcT*), which mediates GlcCer synthesis. However, flies do not produce SM but use CPE as the major SL component in the plasma membrane (Rietveld et al., 1999). CPE is also utilized in flies to perform similar roles of GalCer in the glial cells ensheathing the peripheral axons (Schwann cells in human and wrapping glia in flies) (Ghosh et al., 2013). The higher order GSLs in flies also differ from the human counterparts in that they are built on mannosyl glucosylceramide (MacCer) rather than LacCer. However, expression of human *B4GALT6* gene, which encodes a LacCer synthase, rescues the loss of the fly MacCer synthase gene *egh*, arguing that the functions of the GSLs in human and flies are comparable (Wandall et al., 2003, 2005).

### SL catabolism—SM pathway and salvage pathway

Besides *de novo* synthesis, Cer can also be produced by the hydrolysis of complex SLs. The catabolic pathways occur at two locations, the plasma membrane and the lysosomes. On the plasma membrane, some complex SLs, such as SM, can be hydrolyzed into Cer by neutral hydrolases (also known as SM pathway) (Figure 2, bottom right). Alternatively, sections of the plasma membrane can be endocytosed *via* the endolysosomal pathway, and the complex SLs in the membrane are hydrolyzed in the lysosomes by the acidic enzymes (also known as salvage pathway) (Figure 2, top right). Among the complex SLs, SM and GlcCer can be transformed into Cer by both pathways, while the hydrolysis of CPE, GalCer and higher order GSLs occurs exclusively in lysosomes. At both locations, Cer can be further hydrolyzed into Sph by ceramidase, which can feed into the SL degradation pathway (described below).

In flies, GlcCer can be hydrolyzed by both acidic and neutral glucosylceramidase. The pathway mediating CPE hydrolysis, however, is not well studied. Mammals only produce trace amount of CPE (Bickert et al., 2015). While no specific CPE hydrolase has been identified in humans, human SMPD1 (acidic sphingomyelinase) is able to use CPE as an alternative substrate (Breiden and Sandhoff, 2021). Flies have three predicted orthologs of *SMPD1* and one ortholog of *SMPD2* (neutral sphingomyelinase), but the activity of these enzymes has not been studied. For Cer hydrolysis, flies have an ortholog of neutral ceramidase (*ASAH2*) but not acidic ceramidase (*ASAH1*), indicating that the hydrolysis of Cer primarily occurs on the plasma membrane in flies.

### S1P-mediated SL degradation

Cer can be converted to Sph upon hydrolysis, as mentioned above. Sph is then phosphorylated to make Sph-1-phosphate (S1P) and degraded into PE and fatty aldehyde by the S1P lyase. S1P can be hydrolyzed back into Sph by S1P phosphatases (Mandala, 2001), while the degradation of S1P by S1P lyase is irreversible. The S1P-mediated degradation pathway is highly conserved between human and flies.

## Modeling neurodegenerative diseases using *Drosophila*

Fruit flies have a sophisticated nervous system which has numerous similarities to the vertebrate nervous system. Its sensory modalities include vision (Joly et al., 2016; de Andres-Bragado and Sprecher, 2019), hearing (Albert and Gopfert, 2015), olfaction (Ramdya and Benton, 2010), taste (Yarmolinsky et al., 2009), thermosensation (Barbagallo and Garrity, 2015), mechanosensation (Tuthill and Wilson, 2016), proprioception (Agrawal and Tuthill, 2022), and nociception (Im and Galko, 2012; Gibbons et al., 2022). The motor system is also sophisticated and allows fine control of motor activities including gait and flight (Clark et al., 2018; DeAngelis et al., 2019; Zarin et al., 2019; Phelps et al., 2021). The fly nervous system also has complex sensory-motor circuits and the neuronal networks permit high order functions including complex behavioral outputs, including learning, and memory (Frye, 2010; Pool and Scott, 2014; Auer and Benton, 2016; Cognigni et al., 2018; Modi et al., 2020). At the cellular level, flies contain many neuron subtypes that use 10 out of 11 classes of neurotransmitters present in human, including -aminobutyric acid (GABA), glutamate, acetylcholine, and dopamine. Moreover, compelling data argue that glial cells in flies are functionally analogous to human astrocytes, oligodendrocytes, and Schwann cells (Freeman and Doherty, 2006; Deng et al., 2019). Recent single cell transcriptomics studies identified ∼90 distinct neuronal cell types in fly heads, again implying a high level of complexity of the fly nervous system (Li et al., 2022; Lu et al., 2022).

The similarities at the molecular, cellular, and functional levels between the human and fly nervous system empower the study of gene function in flies and permit comparisons with vertebrate nervous systems (Ugur et al., 2016; Ma et al., 2022). At the subcellular level, defects in organelles like ER, mitochondria and lysosomes are commonly observed in NDDs, which are readily detected in flies using molecular markers or transmission electron microscopy (TEM). These changes, as well as the morphological changes in neurons/glia, can be easily assessed in numerous neuronal tissues (Table 2). At the organismal level, a battery of fly behavioral assays enables the assessment of defects associated with neuronal dysfunction. These include shortened lifespan, defects in climbing and flight, susceptibility to seizures induced by mechanical and heat stress, aberrant circadian rhythms and sleep, impaired vision, olfaction, learning and memory, as well as defects that can be assayed with numerous other behavioral paradigms that are used less frequently (Table 2).

**TABLE 2:** Assays to study neurodegeneration in *Drosophila.*

Assays	Descriptions	Related defects in human	References
Lifespan	Measurement of the lifespan of flies from eclosion to death	Reduced lifespan	Lin et al., 2018; Lu et al., 2022; Srivastava et al., 2023
Climbing and flight activity	Measurement of the anti-geotaxis climbing and flying activities of adult flies	Motor defects	
Bang and heat sensitivity	Measurement of the time to recover from a vortex induced shaking or 42°C heat shock	Seizures	
Circadian rhythm and sleep	Evaluation of the circadian rhythm and sleep by continuous monitoring of locomotion activity using a *Drosophila* activity monitoring system	Defects in circadian rhythm and sleep	Pfeiffenberger et al., 2010; Wangler et al., 2017
Vision and olfaction	Evaluation of the vision and olfaction of flies using photo-taxis and odor-taxis assays	Vision or olfactory loss	Ali et al., 2011; Kahsai and Zars, 2011; Mariano et al., 2020
Learning and memory	Evaluation of the learning and memory capacity of odors, visual patterns, and spatial cues using conditioning assays	Learning disability, memory loss	
Neuromuscular junction (NMJ) (Morphology and electrophysiology)	Measurement of the number, distribution, size, and morphology of synaptic boutons at the NMJs, and precise measurement of the physiological properties of synaptic transmission	Neuromuscular defects	Oswald et al., 2015
Motor neuron axons (Morphology)	Measurement of the structure integrity and axonal transport of the long axon extended from the ventral nerve cord to the muscles	Axonal defects in motor neurons	Yalcin et al., 2017
Wing margin nerves (Morphology)	Measurement of the degeneration of axons and wrapping glia	Axonal defects and demyelination	Chung et al., 2020
Compound eye (ERG)	Measurement of the neuronal activity triggered by light stimulation	Functional abnormalities in retina and other neurological defects	Wang et al., 2014, 2022c; Ye et al., 2020
Compound eye (Histology)	Observation of the pathological changes in ommatidia using histological staining, fluorescent markers and immunolabeling	Cellular and subcellular neurodegenerative changes	
Compound eye (TEM)	Observation of the ultrastructural and organelle changes in ommatidia		

Of note, the fly eye has been a very valuable model to study the molecular and cellular mechanisms underlying numerous neurological disorders. The compound eye consists of ∼800 independent photosensing units named ommatidia. Every ommatidium is comprised of photoreceptor neurons, glia, and other cell types organized in a highly stereotyped pattern, allowing identification of all the different cell types (Perry et al., 2017). Second, one can induce small to large homozygous mutant clones in the eye and hence often avoid issues associated with lethality caused by the loss of essential genes (Wang et al., 2014; Yamamoto et al., 2014; Chen et al., 2016b). Third, the repetitive pattern of ommatidia allows detection of subtle phenotypes and comparison between neighboring wildtype and mutant ommatidia. Fourth, neuronal activity in the eye can be tightly controlled by modulating light intensity, which is a unique and important feature as it is difficult to continuously control neuronal activity in other neurons (Wang et al., 2014, 2022c). Fifth, neuronal activity can be easily and precisely measured using electroretinography (ERG). Sixth, the detailed cellular morphology can easily be visualized by TEM to detect cellular and especially organellar defect (Lin et al., 2018; Wang et al., 2022c). Finally, genetic approaches allow manipulations of different neuronal and glial cells which facilitates the study the function of genes in different cells as well as neuron-glia interactions (Liu et al., 2015, 2017; Moulton et al., 2021; Wang et al., 2022c). The unique anatomical and physiological characteristics of the fly eye combined with numerous tools allow in depth studies that cannot be easily achieved in most models. Although the fly eye does not recapitulate all the features of other neurons and glia, the wealth of knowledge obtained in the fly eye has very often proved true in other areas of the nervous system, especially with respect to the study of the mechanism of neurodegeneration.

## Inherited peripheral neuropathies

Neurodegenerative diseases of the peripheral nervous system (PNS) can be classified into motor neuropathies, motor and sensory neuropathies, and sensory and autonomous neuropathies based on the nature of the affected neurons. Among these many are based on a monogenic inheritance pattern, and recent findings established an intriguing link between the genes encoding subunits of SPT, the rate limiting enzyme in the SL *de novo* synthesis pathway, and three inherited peripheral neuropathies.

### Hereditary sensory and autonomous neuropathy 1

Hereditary sensory and autonomous neuropathy (HSAN) refers to a group of neurodegenerative disorders with heterogenous clinical features with sensory, autonomous and minor motor neuron involvement. HSAN1 is one of five HSAN subtypes. It is inherited in an autosomal dominant (AD) fashion by mutations in one of five genes: *SPTLC1* (Dawkins et al., 2001; Bode et al., 2016; Gantner et al., 2019), *SPTLC2* (Rotthier et al., 2010; Bode et al., 2016; Gantner et al., 2019), *ATL1* (Guelly et al., 2011), *ATL3* (Kornak et al., 2014), and *DNMT1* (Klein et al., 2011). The symptoms include loss of pain and temperature sensation that initiate in the distal section of the lower limbs and progressively spread proximally. In addition, variable motor and autonomous symptoms present in some patients.

The clinical features of *SPTLC1-* (OMIM# 162400) and *SPTLC2*-associated HSAN1 (OMIM# 613640) are very similar, indicating a common disease-causing mechanism. Variants in both *SPTLC1* and *SPTLC2* disrupt the substrate selectivity for L-serine vs. L-alanine. This causes an aberrant reaction with palmitoyl-CoA. The product of the L-alanine/palmitoyl-CoA condensation is 1-deoxysphinganine (doxSA), which cannot be further processed into complex SLs by the downstream enzymes. Therefore, doxSA accumulates in cells and causes neurotoxicity (Gable et al., 2010; Penno et al., 2010). Based on cryo-EM 3D structure of the SPT enzyme complex, all the HSAN1 variants map to the active site of the enzyme complex surrounding the SPTLC1-SPTLC2 interacting interface, supporting that this is a critical disease mechanism (Li et al., 2021; Wang et al., 2021).

*SPTLC1/2*-associated HSAN1 has been modeled in mammals and flies. In a mouse model, a disease-causing *Sptlc1*^*C*133*W*^ variant was overexpressed ubiquitously and shown to cause doxSA production. However, the mice display no motor phenotype and very mild, age-dependent sensory phenotypes at 8–10 months. Despite this mild phenotype, the ectopic expression of Sptlc1^C133W^ causes demyelination of axons and axonal damage in the PNS (McCampbell et al., 2005). Recently, a novel *Sptlc1*^*C*133*W*^ knock-in mice model was generated. The 12-month-old *Sptlc1*^*C*133*W*/+^ mice display aberrant doxSA production, mild sensory and motor defects, and no sign of axon degeneration (Hines et al., 2022).

*Spt-I* is the fly ortholog of *SPTLC1*. A fly HSAN1 model was generated by overexpressing a *Spt-1* variant analogous to human *SPTLC1*^*C*133*W*^ variant (*Spt-I*^*C*129*W*^) under control of the GAL4/UAS system (Oswald et al., 2015). UAS-*Spt-I*^*C*129*W*^ was expressed ubiquitously (*tub-Gal4*) or specifically in a subset of peripheral sensory neurons (*ppk-Gal4*). *Spt-I*^*C*129*W*^ expression in the sensory neurons induces a sensory deficit in a heat avoidance assay, which is obvious in larvae. Ubiquitous *Spt-I*^*C*129*W*^ expression compromises viability to adulthood but only causes a slight, non-significant overproduction of deoxysphingoid bases. Importantly, feeding flies with a dietary L-alanine supplement significantly elevates the deoxysphingoid base production and exacerbates the toxicity of the transgene. In contrast, dietary L-serine alleviates the defects caused by the transgene, indicating a possible therapeutic strategy (Oswald et al., 2015). Dietary L-serine supplement was also tested in the *Sptlc1*^*C*133*W*^ overexpression mouse model as well as in patients with the *SPTLC1*^*C*133*Y*^ variant. The treatment significantly lowers the level of doxSA in mice tissues and patient plasma. The symptoms in mice are significantly improved by L-serine treatment (Garofalo et al., 2011). Following this study, a clinical trial was carried out which shows that oral L-serine supplementation appears safe in patients and is potentially effective at slowing disease progression (Fridman et al., 2019).

### Juvenile-onset amyotrophic lateral sclerosis

Amyotrophic lateral sclerosis is a motor neuron disease with an incidence between 0.6 and 3.8 per 100,000 person per year (Longinetti and Fang, 2019). The disease is characterized by progressive degeneration of both upper and lower motor neurons (UMNs and LMNs). While most of the sporadic cases show a late onset (between 51 and 66 years in average) (Longinetti and Fang, 2019), patients with familial inherited ALS can present with symptoms earlier. The initial clinical presentations of ALS vary among individuals. In some individuals the disease onset is in the limbs, characterized by a combination of UMN (spasticity, weakness, and increased deep tendon reflexes) and LMN signs (muscle twitching, wasting, weakness). Others have a bulbar onset, with speech issues (dysarthria) and swallowing difficulties (dysphagia). In either case, the disease is relentlessly progressive, and most cases ultimately develop severe dysphagia and respiratory insufficiency which cause malnutrition and ultimately death. Currently the management of the disease largely involves symptomatic treatments. Although the majority of the ALS cases do not have discernible family history, a number of monogenic subtypes of ALS have been identified (Goutman et al., 2022) and many of the ALS disease-causing genes have been modeled in organisms including fruit flies (Azuma et al., 2018).

Changes in SL levels have been assessed in ALS patient samples and mice models, but results vary depending on the tissue type and animal models tested. Elevated levels of Cer, SM, and GSLs are observed in patient spinal cord samples, indicating an association between SL and ALS (Cutler et al., 2002; Dodge et al., 2015). However, SL data of serum and CSF in different studies are not consistent (Blasco et al., 2017; FernAndez-Eulate et al., 2020; Goutman et al., 2020; Area-Gomez et al., 2021; Sol et al., 2021). Interestingly, *wobbler* mice which are considered a model for ALS and carry a partial loss-of-function mutation of *Vps54* (Moser et al., 2013), have elevated SL levels in embryonic fibroblasts and increased Sph levels in the spinal cord. Treatment with myriocin, an inhibitor of the SPT complex, improves motor activity and neuropathological changes, suggesting that an accumulation of SL contributes to the progression of disease (Petit et al., 2020).

Recently, two independent studies reported patients with *SPTLC1* variants who present with a juvenile-onset ALS (Johnson et al., 2021; Mohassel et al., 2021), providing a direct link between sphingolipid metabolic dysfunction and the pathogenesis of ALS. Unlike the *SPTLC1* variants associated with HSAN1, the ALS *SPTLC1* variants map to the ER transmembrane domain of SPTLC1 where it interacts with ORMDL protein (Li et al., 2021; Wang et al., 2021). ORMDL suppresses SPT activity in the presence of excessive ceramide in the ER (Siow and Wattenberg, 2012; Gupta et al., 2015; Davis et al., 2019). The variants dominantly disrupt the interaction between SPTLC1 and ORMDL protein and cause an increase in SL *de novo* synthesis (Johnson et al., 2021; Mohassel et al., 2021). Furthermore, Mohassel et al. (2021) showed that allelic-specific siRNA effectively reduced the mutant mRNA levels and the SL levels in patient derived fibroblasts. So far, no animal model of the *SPTLC1*-associated ALS has been reported and future efforts are needed to elucidate the mechanism by which elevated SL synthesis causes degeneration of the motor neurons.

### Hereditary spastic paraplegia

Hereditary spastic paraplegia (HSP) refers to a group of motor neuron disease that are characterized by the progressive spasticity and weakness in the lower extremities. Unlike ALS which affects both UMNs and LMNs, HSP only involves the degeneration of LMNs. Clinically the disease can be classified into a pure form and a complex form, depending on the existence of complications other than the LMN signs. Nearly all the HSP subtypes show monogenic inheritance and more than 80 genes have been associated with the disease (Panza et al., 2022). Depending on the affected gene, the disease can be inherited in AD, autosomal recessive (AR), X-linked recessive (XR), or *via* a mitochondrial inheritance pattern. The age of disease onset also varies among different disease subtypes.

The involvement of sphingolipid metabolism in the progression of HSP recently surfaced. Variants in *SPTSSA* cause an early onset and complex form of HSP. Individuals with *SPTSSA* variants were reported to develop spasticity and weakness in lower limbs as well as epilepsy, axial hypotonia and sensorineural hearing loss. SPTSSA is an activating subunit in the SPT complex. The disease-causing variants affect the C-terminus of SPTSSA protein, where it interacts with the ORMDL protein. Similar to the ALS variants in the *SPTLC1* gene, the *SPTSSA* variants disrupt the interaction between SPTSSA and ORMDL and lead to increased SPT activity and SL *de novo* synthesis in human cells (Srivastava et al., 2023).

To study the phenotypes associated with increased SL synthesis *in vivo*, a fruit fly model was established by overexpressing the three human SPT subunits as a fusion protein. Expression of human SPT in flies leads to excessive SL synthesis and causes severe motor defects as well as a shortened lifespan. However, co-expressing human ORMDL3 fully rescues the defects caused by the reference SPT showing that the human enzyme complex is functional and properly regulated in flies. The SPT fusion protein with the *SPTSSA*^*T*51*I*^ variant causes similar defects as expression of the reference SPT, showing that the missense mutation does not alter enzymatic activity. However, *SPTSSA*^*T*51*I*^ activity is only partially suppressed by expression of ORMDL3 leading to elevated levels of Cers, motor defects, and shorter lifespan (Srivastava et al., 2023). The model was also used to test a mice *Sptssb*^*H*56*L*^ variant which causes increased SL *de novo* synthesis and neurodegeneration in *Stellar* mice (Zhao et al., 2015). Similar to the *SPTSSA*^*T*51*I*^** variant, the *SPTSSA*^*H*59*L*^ (*Sptssb*^*H*56*L*^ analog) also disrupts the SPTSSA-ORMDL interaction and causes neurological phenotypes in flies (Srivastava et al., 2023). These data establish that the SPTSSA variants found in three individuals cause elevated Cer levels because they fail to be properly regulated by ORMDL.

## Lysosomal storage diseases

Lysosomal storage diseases (LSDs) are a group of inherited metabolic disorders that affect lysosomal functions [for a comprehensive review of LSDs, see (Platt et al., 2018)]. Most LSDs are caused by variants in genes that encode lysosomal proteins required for lysosomal catabolism. The loss-of-function of the affected protein results in the accumulation of substrates in lysosomes and ultimately causes a severe cell dysfunction. LSDs are clinically heterogeneous, but most LSDs present with early onset neurodegenerative features. Interestingly, many LSD genes have been implicated as risk factors in PD (Robak et al., 2017; Blauwendraat et al., 2020a).

Accumulation of SL species (sphingolipidosis) has been documented for several LSDs (Table 3), including those associated with genes encoding SL salvage pathway enzymes as well as the *PSAP* gene. *PSAP* encodes the prosaposin protein, the precursor of four saposin proteins (saposins A–D) which facilitate the hydrolysis of GSLs in lysosomes (O’Brien and Kishimoto, 1991). Besides the SL-related LSDs, sphingolipidosis is also observed in other LSDs that are not directly associated with SL-related genes. Niemann-Pick Disease type C (NPC, OMIM# 257220, 607625) is associated with *NPC1* and *NPC2* genes that encode lysosomal cholesterol transporter proteins. Variants in these genes cause a lysosomal accumulation of cholesterol as well as various SL species including Sph, SM and GSLs (Newton et al., 2018). Secondary accumulations of gangliosides (one subtype of GSL) are observed in a wide spectrum of LSDs, and are not limited to those involving primary defects in ganglioside degradation (Walkley, 2004).

**TABLE 3:** Lysosomal storage disorders associated with lysosomal sphingolipid accumulation.

Disease genes	Protein	Substrate accumulated	Disease	OMIM#
*ASAH1*	Ceramidase	Cer	Farber disease	228000
*GBA*	Glucosylceramidase	GlcCer	Gaucher disease	230800, 230900, 231000, 231005, 608013
*GALC*	Galactosylceramidase	GalCer	Krabbe disease	245200
*SMPD1*	Sphingomyelinase	SM	Niemann-pick disease A/B	257200, 607616
*ASRA*	Arylsulfatase A	Sulfatide	Metachromatic leukodystrophy	250100
*GLA*	α-galactosidase	Gb3 globosides	Fabry disease	301500
*GLB1*	β-galactosidase	GM1 gangliosides	GM1-gangliosidosis	230500, 230600, 230650
*HEXA*	β-hexosaminidase	GM2 gangliosides	Tay-sachs disease	272800
*HEXB*	β-hexosaminidase	GM2 gangliosides	Sandhoff disease	268800
*PSAP*	Saposin A-D	Multiple GSLs	Combined SAP deficiency	611721
	Saposin A	GalCer	Krabbe disease, atypical	611722
	Saposin B	Sulfatide	Metachromatic leukodystrophy	249900
	Saposin C	GlcCer	Gaucher disease, atypical	610539
*NPC1*	NPC1	Cholesterol, Sph, SM, GSLs, etc.	Niemann-pick disease C	257220
*NPC2*	NPC2			607625

Limited by the scope of this review, we will only discuss two LSDs that are well-modeled in fruit flies: Gaucher disease (GD) and combined saposin deficiency. Though Niemann-Pick Disease type C has been modeled in fruit flies, the changes in SL metabolism were not assessed and therefore will not be discuss here (Huang et al., 2007; Phillips et al., 2008).

### Gaucher disease

Gaucher disease is a rare, AR disease caused by variants in *GBA* (Mistry et al., 2017; Grabowski et al., 2021). *GBA* encodes a lysosomal glucosylceramidase (GCase, also called glucocerebrosidase) which facilitates hydrolysis of GlcCer into Cer. Deficiency of GCase activity results in accumulation of the substrate GlcCer. Classically, the disease is classified into three subtypes: type I (non-neuronopathic; OMIM# 230800), type II (acute neuronopathic; OMIM# 230900), and type III (subacute neuronopathic; OMIM# 231000, 231005), depending on the presence of neurological symptoms and the age of onset. The clinical presentations among subtypes are highly variable and form a continuum. Type II GD patients present in infancy with progressive brainstem dysfunction, seizures and other neurological deficits. Type III GD patients present with similar manifestations but have a later onset (from childhood to adult) and the disease progresses more slowly (Roshan Lal and Sidransky, 2017). Although type I GD is classified as non-neuronopathic, neurological symptoms have been reported in a fraction of type I GD patients as the disease progresses (Biegstraaten et al., 2008; Capablo et al., 2008). In addition to the direct association with GD, *GBA* has also been identified as a risk factor for PD (see section “Parkinson’s disease and parkinsonism”).

*Drosophila* has two orthologs of *GBA*, *Gba1a*, and *Gba1b*. *Gba1a* is expressed in the fly gut, while *Gba1b* is more broadly expressed (Davis et al., 2016). Several *Gba1b* mutant fly models have been established to study neuronopathic GD (Davis et al., 2016; Kinghorn et al., 2016; Thomas et al., 2018; Wang et al., 2022c). The *Gba1b* null mutants exhibit a 16-fold GlcCer accumulation in the head and have a short lifespan, progressive motor defects, and memory defects when compared to controls. In the fly CNS, synaptic loss and neurodegeneration is obvious. At the cellular level, the loss of *Gba1b* results in the accumulation of p62/Ref(2)P-containing protein aggregates, lysosomal expansion and disrupted ATP production in mitochondria (Davis et al., 2016; Kinghorn et al., 2016). Importantly, a recent study found that Gba1b is enriched in glial cells, but not in neurons. In flies increased neuronal activity induces GlcCer production in neurons. The GlcCer is secreted by neurons *via* exosomes, taken up by glia where it is degraded in lysosomes. This process is triggered by glial-derived TGF-β/BMP signal (Wang et al., 2022c). Intercellular GlcCer transport also occurs in mammalian cells and mammalian glial cells express a significantly higher level of *GBA* than do neurons, providing evidence for the conservation of the GlcCer transfer from neurons to glia in mammals (Wang et al., 2022c). The findings support an important role for glial cells in the progression of neuronopathic GD.

### Saposin deficiencies

Saposins are a group of proteins that facilitate GSL hydrolysis in the lysosomes [for review, see (O’Brien and Kishimoto, 1991)]. A single gene, *PSAP*, encodes the precursor of saposins (prosaposin), which is further processed in endosomes into four proteins (Saposin A–D). All saposin proteins are non-enzymatic activators of lysosomal GSL hydrolases (O’Brien and Kishimoto, 1991; Azuma et al., 1994). Saposin-A and C activate glucosylceramidase and galactosylceramidase. Saposin-B activates arylsulfatase A, α-galactosidase and β-galactosidase. Last, Saposin-D activates acid sphingomyelinase and ceramidase. Variants in the *PSAP* gene cause four distinct disorders depending on the domain(s) of the prosaposin protein affected. Deficiency in one single saposin protein causes Krabbe- (Saposin-A), metachromatic leukodystrophy- (Saposin-B) or Gaucher-like phenotypes (Saposin-C). Variants affecting multiple saposins cause a combined saposin deficiency which causes a sphingolipidosis with very early onset and severe neurological deficits (Harzer et al., 1989; Hulkova et al., 2001; Kuchar et al., 2009). One study reported that variants affecting Saposin-D cause an AD form of parkinsonism (PARK24, OMIM# 619491) in three families and that *PSAP* intronic variants near the Saposin-D domain-coding exons are a risk factor for sporadic PD (Oji et al., 2020b). Unexpectedly, no significant loss of SL hydrolase activity was observed in the patient-derived cells (Oji et al., 2020b) and follow-up studies reported that *PSAP* variants are rarely identified in large PD cohorts (Facchi et al., 2020; Oji et al., 2020a, 2021a,b,c; Sosero et al., 2020; Chao et al., 2021; Lin et al., 2021; Zhao et al., 2021).

The combined saposin deficiency has been modeled using fruit flies. The fruit fly has one ortholog of *PSAP*, *Sap-r*. *Sap-r* mutants have a short lifespan, a progressive decline in locomotor activity and a progressive decline in neuronal activity in photoreceptors. *Sap-r* loss causes an age-dependent vacuole formation and neurodegeneration in the adult brain. It also induces autophagy, lysosome expansion and mitochondrial dysfunction in multiple tissues based on transmission electron microscopy. Lipidomic profiling revealed a progressive sphingolipidosis including Cer, GlcCer, CPE and higher order GSLs (Hindle et al., 2017; Sellin et al., 2017). Similar complex sphingolipidosis are observed in combined saposin deficiency patients and *SAP* knockout mice models (Bradova et al., 1993; Fujita et al., 1996; Oya et al., 1998; Hulkova et al., 2001; Kuchar et al., 2009).

## Friedreich’s ataxia

Friedreich’s ataxia (FRDA, OMIM# 229300) is an AR neurodegenerative disorder that affects 1 in every 40–50,000 children worldwide. Typically, the symptoms start to develop between the ages of 10–15, and patients present with gait issues and ataxia, dysarthria, muscle weakness, and cardiomyopathy (Cook and Giunti, 2017; Indelicato et al., 2020). Patients also suffer from diabetes mellitus, scoliosis, and late-onset optic neuropathy (Fortuna et al., 2009; Noval et al., 2012; Cook and Giunti, 2017). More than 95% of FRDA are caused by a homozygous triplet repeat expansion of GAA in the first intron of the *FXN* gene. *FXN* encodes a mitochondrial protein Frataxin. Less than 5% of the FRDA patients are compound heterozygous for *FXN*, where one allele contains a point mutation and the other the repeat expansion in the first intron. Notably, the age of onset is directly correlated with the GAA repeat numbers and protein levels (Indelicato et al., 2020). In healthy individuals, the first intron of *FXN* contains <40 GAA repeats whereas the repeat number increases up to >1000 in individuals with FRDA (Cook and Giunti, 2017; Kelekci et al., 2022). Increased GAA repeat numbers in *FXN* reduces the protein levels to 4–29% of normal levels, providing supporting evidence that loss of Frataxin is the cause for the disease (Campuzano et al., 1997). Frataxin is required for iron-sulfur (Fe-S) cluster assembly in mitochondria. Fe-S clusters act as cofactors for aconitase and functions together with the enzymes of the electron transport chain (ETC) in mitochondria as well as in regulation of iron metabolism (Maio and Rouault, 2020). Indeed, loss of *FXN* leads to iron deposition in the brain and cardiomyocytes in FRDA patients (Lamarche et al., 1980; Waldvogel et al., 1999; Michael et al., 2006; Ward et al., 2019).

Similar phenotypes including mitochondrial abnormalities, iron accumulation and progressive neurodegeneration have been noted in fruit fly FRDA models. Ubiquitous knockdown of fruit fly *frataxin* (*fh*) causes developmental lethality whereas neuronal or glial knockdown reduces lifespan and impairs motor activity (Anderson et al., 2005; Navarro et al., 2010; Monnier et al., 2018). In addition, loss of glial *fh* leads to glial lipid droplet accumulation and ubiquitous knockdown leads to elevated levels of fatty acids (Navarro et al., 2010). Chen et al. (2016a),b characterized the first fly *fh* mutant and discovered an increase in ceramide levels in flies as well as in FRDA patient-derived cardiac samples. Both in *fh* mutants as well as in patient heart samples, the levels of dhSph, Sph, dhCer, Cer were increased and a recent study also reported elevated ceramide levels in FRDA patient-derived fibroblasts (Wang et al., 2022b). These observations point to a connection between mitochondrial dysfunction, elevated iron levels and elevated Cer levels. However, how impaired mitochondrial function and/or iron metabolism elevates Cer levels in FRDA patients has not yet been explored. Interestingly, some of the SL metabolic enzymes, namely ceramide synthase, ceramidase, sphingomyelinase and SPTLC2, have been reported to be present in mitochondria (Roszczyc-Owsiejczuk and Zabielski, 2021; Aaltonen et al., 2022). Further studies are required to determine how mitochondrial iron dyshomeostasis increases the levels and/or activity of these enzymes and contribute to the elevated ceramide levels in FRDA.

## S1P-associated disorders

### Acyl-CoA oxidase deficiency

Peroxisomes are subcellular organelles that are involved in metabolic processes including the β-oxidation of very long chain fatty acids (VLCFA) (Singh et al., 1984). Acyl-CoA oxidase 1 (ACOX1) is the first and rate-limiting enzyme in the fatty acid β-oxidation of VLCFA (Fournier et al., 1994). The oxidase activity of ACOX1 produces hydrogen peroxide (H_2_O_2_) as a byproduct (Schrader and Fahimi, 2006). AR mutations in *ACOX1* are associated with acyl-CoA oxidase deficiency (OMIM# 264470). Patients with acyl-CoA oxidase deficiency are reported to develop a rapid and severe loss of function in the nervous system, characterized by hypotonia, seizures, visual system failure, white matter abnormalities, inflammatory responses and loss of motor achievements (Ferdinandusse et al., 2007).

*Drosophila dACOX1*, the ortholog of human *ACOX1*, is expressed mostly in glia and absent in neurons of the central nervous system (Chung et al., 2020). Loss of *dACOX1* leads to increased VLCFA levels, glial cell death, reduced neuronal survival, and shortened lifespan in flies (Chung et al., 2020). In addition, lack of *dACOX1* specifically in glia leads to elevated levels of Cers with VLCFA which are highly enriched in membranes of cells that wrap around axons, similar to Schwann cells in vertebrates and wrapping glia in flies (Chung et al., 2022). Interestingly, elevated levels of VLCFA-Cers in glia are not toxic in flies but they lead to the production of elevated levels of S1P in glia, which is toxic (Chung et al., 2022). S1P regulates diverse cellular processes and induces immune responses and inflammation (Spiegel and Milstien, 2011). Moreover, S1P is produced and released from glia and taken up by neurons in flies and elevated levels of S1P causes an activation of the immune deficiency pathway (IMD) and an invasion of immune cells in the brain. Neuronal expression of either the fly gene (*sply*) or the human gene (*SGPL1*) encoding S1P lyase significantly rescues the motor defects caused by glial overproduction of S1P, showing that S1P is toxic in neurons (Chung et al., 2022). Other studies have shown that S1P prevents ceramide-induced apoptosis in non-neuronal, non-glial cells (Cuvillier et al., 1996; Osawa et al., 2001; Castillo and Teegarden, 2003), indicating cell-type specificity of S1P activity. Interestingly, drugs that lower VLCFA synthesis or inhibit the action of S1P are highly beneficial in flies that lack *ACOX1* as well as in mouse model for Multiple Sclerosis (Chung et al., 2022). Hence, a specific population of ceramides with VLCFA may be at the root of elevated S1P synthesis when Schwann cells or oligodendrocytes are affected in some NDDs.

### SGPL1-associated Charcot-Marie-Tooth disease

Sphingosine-1-phosphate lyase 1 (SGPL1), an ER-localized enzyme encoded by the *SGPL1* gene, catalyzes the final step of S1P breakdown (Prasad et al., 2017). SGPL1 deficiency causes an AR, axonal form of Charcot-Marie-Tooth disease (CMT). CMT represents a heterogeneous group of peripheral motor and sensory neuropathies. Only two probands with *SGPL1*-associated CMT have been identified thus far. Probands present with a juvenile-onset muscle weakness, muscle wasting, and a progressive decrease of motor neuron conduction velocity. A mild sensory deficit is observed in one of the two probands. Increased levels of S1P, as well as Sph/dhSph, are observed in the plasma of both probands (Atkinson et al., 2017). Although the toxicity of S1P accumulation is not directly demonstrated, the case report is suggestive of a role of S1P accumulation in neurodegeneration.

The *Drosophila* ortholog of *SGPL1* is *Sply*. Loss of *Sply* causes semi-lethality and increased apoptosis in developing embryos. The surviving flies display an abnormal flight muscle morphology (Herr et al., 2003). Blocking the S1P synthesis by a sphingosine kinase inhibitor, D, L-threo-DHS, partially rescues the phenotypes of *Sply* mutants, suggesting that the muscle phenotype of *Sply* mutants is caused by S1P accumulation (Herr et al., 2003). Specific knockdown of *Sply* in neurons caused impaired arborization and reduced synaptic bouton number at the neuromuscular junction (NMJ) as well as degeneration of the sensory neurons in the wing blades, further indicating the toxicity of S1P accumulation in neurons (Atkinson et al., 2017).

## Parkinson’s disease and parkinsonism

Parkinson’s disease is a common neurodegenerative disease with an increasing prevalence in the aging population (de Lau and Breteler, 2006). The disease is primarily defined by core motor symptoms including bradykinesia, rest tremor and rigidity. It can also cause a wide spectrum of non-motor symptoms, such as cognitive impairment, autonomic dysfunction and sleep disorders (Schapira et al., 2017). The loss of dopaminergic (DA) neurons in the *substantia nigra pars compacta* is commonly observed in patients, leading to disruption of nigrostriatal pathway and progression of motor dysfunctions (Giguere et al., 2018). A pathological hallmark of PD is the accumulation of α-synuclein (α-Syn) that results in the formation of proteinaceous cytoplasmic inclusions known as Lewy bodies and Lewy neurites (Sulzer and Edwards, 2019). The existing treatments for PD motor symptoms are primarily dopamine based, which decelerate the disease progression but do not modify the pathogenesis (Armstrong and Okun, 2020). Disease-modifying α-Syn targeting therapies have also been proposed (Fields et al., 2019; Fleming et al., 2022), but none has been approved by the FDA in the US. So far, there is no cure for PD.

Historically, PD was considered a sporadic disease until the identification of *SNCA* variants that caused monogenic inheritance of PD (Polymeropoulos et al., 1997). In the past 2.5 decades, our understanding of the genetics of PD has vastly improved (Vazquez-Velez and Zoghbi, 2021; Ye et al., 2022). Numerous Mendelian inherited PD subtypes and the identification of ∼100 risk genes/loci *via* genome-wide association studies (GWAS) have provided potential clues as to what triggers PD in some individuals (Nalls et al., 2019; Blauwendraat et al., 2020a; Guadagnolo et al., 2021). Highly penetrant, rare variants of the known PD-causing genes account for 10–15% of all the PD cases (Verstraeten et al., 2015). In other cases, the disease is associated with disease-causing variants with incomplete penetrance, such as those in the *LRRK2* and *GBA* genes (Healy et al., 2008; Sidransky et al., 2009; Milenkovic et al., 2022; Rocha et al., 2022). New insights have also been driven by the discovery of genetic modifiers and oligogenic etiology of PD (Lubbe et al., 2016; Robak et al., 2017; Rousseaux et al., 2018; Bandres-Ciga et al., 2020; Blauwendraat et al., 2020b; Iwaki et al., 2020; Ren et al., 2022; Straniero et al., 2022).

The linkage between SL dysmetabolism and PD was first implicated by the increased incidence of PD in GD patients and individuals carrying a single *GBA* variant. Patients with type I GD have a 20-fold increased risk of developing PD when compared to the general population (Bultron et al., 2010). Further, the presence of a single variant of *GBA* increases the PD risk by a factor of 5 (Sidransky et al., 2009). In addition to *GBA*, at least three other SL-related LSD genes have been identified as PD risk factors, including *SMPD1*, *ASAH1*, and *PSAP* (Robak et al., 2017; Oji et al., 2020b). In non-*GBA* PD cohorts or cohorts without genotype information, lipidomics data have not revealed consistent SL level changes in PD patients vs. healthy controls in postmortem brain tissue, serum and CSF (Custodia et al., 2021; Esfandiary et al., 2022). However, in one *GBA*-PD cohort mild increases in levels of Cer, hexosylceramide (GlcCer and GalCer) and LacCer were observed in serum samples (Guedes et al., 2017). The lack of compelling evidence may be due to the nature of the samples that were studied or the dynamic flux in SLs (Lansbury, 2022). However, a role for SLs in the progression of PD is supported by studies in animal models, mostly in fruit flies. Here, we will mainly focus on the fruit fly orthologs of human genes that have been shown to cause PD or parkinsonism and affect SL metabolism. For other PD models in fruit flies, see the following reviews (Hewitt and Whitworth, 2017; Dung and Thao, 2018; Aryal and Lee, 2019).

### SNCA and α-synuclein

Missense and copy number variants of the *SNCA* gene cause AD forms of PD, PARK1 (OMIM# 168601) and PARK4 (OMIM# 605543) respectively. Non-coding *SNCA* variants also increase the susceptibility of PD development (Pihlstrom and Toft, 2011; Deng and Yuan, 2014). The pathogenicity of the *SNCA* copy number variants and the presence of α-Syn containing Lewy Bodies in PD patients lead to multiple efforts to generate α-Syn overexpression transgenic animal models [for review, see (Deng and Yuan, 2014)]. *Drosophila* does not have a *SNCA* ortholog. However, expressing human α-Syn in neurons, either the wildtype protein or the p.A30P and p.A53T pathogenic variants, causes loss of DA neurons and the appearance of α-synuclein positive inclusion bodies in fly brain. This correlates with an age-dependent locomotor dysfunction, however, whether the locomotor defects are caused by DA neuron loss is not determined (Feany and Bender, 2000). Follow up studies further show that DA neuron loss can be induced by specific expression of α-Syn in DA neurons (Auluck et al., 2002; Trinh et al., 2008). Adult specific α-Syn overexpression in fly retina causes marked vacuolization, progressive photoreceptor cell death, and late-onset electroretinogram (ERG) defects (Chouhan et al., 2016). Interestingly, treatment with myriocin, a drug that suppresses SL *de novo* synthesis, suppresses neurodegeneration, indicating that the α-Syn toxicity is, at least partially, mediated by the accumulation of SLs in fly neurons. This is corroborated by a very significant increase in levels of SLs observed in cultured human neurons expressing α-Syn (Lin et al., 2018).

### GBA

The association between *GBA* and PD has been intensely studied in *Drosophila* models. Flies double heterozygous for mutations in *Gba1a* and *Gba1b*, the two orthologs of *GBA*, cause loss of DA neurons, locomotor defects, and a shorter lifespan. These data suggest that GBA heterozygosity may promote PD development, although the direct association between DA neuron loss and other phenotypes is not addressed (Maor et al., 2016). Mutations in *Gba1b* alone does not cause loss of DA neurons in adult fly brains, but the GCase deficiency causes p62/Ref2P-containing protein aggregates that accumulate both in the head and the body of adult mutant flies. GCase deficiency also enhances α-Syn aggregation when α-Syn is expressed in mutant flies, but it does not seem to modify the toxicity of α-Syn (Davis et al., 2016). Yet, Lewy body pathology is commonly observed in GD patients, suggesting an important role of GCase and its substrate GlcCer in the progression of synucleinopathies (Furderer et al., 2022).

The toxicity of α-Syn is due to multiple factors including its misfolding, aggregation, and its propagation across the nervous system (Steiner et al., 2011). Exosome trafficking has been implicated in α-Syn propagation (Danzer et al., 2012; Han et al., 2019). Interestingly, exosomes also mediate the neuron-to-glia trafficking of GlcCer in both flies and human cells (Wang et al., 2022c) and GlcCer facilitates the aggregation of α-Syn (Mazzulli et al., 2011). Collectively, the evidence points to a mechanism by which GCase deficiency promotes PD progression. In this model, accumulated GlcCer promotes α-Syn aggregation which co-propagates with the aggregates *via* exosomes. *Gba1b* mutant flies exhibit a marked increase in exosomes (Thomas et al., 2018), which may exacerbate the pathology. This hypothesis is supported by the findings that pharmaceutical inhibition of GCase leads to an increased number of exosomes containing α-Syn oligomers in mice (Papadopoulos et al., 2018). However, further evidence is required to support this model.

### VPS35

Variants in *VPS35* cause an AD form of PD (PARK17, OMIM# 614203). *VPS35* encodes a core component of the retromer, a complex of three proteins VPS26, VPS29, and VPS35 that mediate the recycling of cargoes from the endosome to the *trans-*Golgi network and the plasma membrane (Small and Petsko, 2015). One of the confirmed disease-causing PD variants, *VPS35^D620N^* (Vilarino-Guell et al., 2011), is a partial loss-of-function variant in various models, indicating that the disease is due to lack of retromer function (Follett et al., 2014; Malik et al., 2015; Ishizu et al., 2016). Single copy loss of *Vps35* in flies does not cause obvious phenotypes, but *Vps35* null mutants are lethal at late larval or prepupal stages (Korolchuk et al., 2007). The loss of *Vps35* in mutant larvae causes structural and functional defects at the NMJ including excessive formation of synaptic terminals, irregular number and size of synaptic vesicles, as well as disrupted neurotransmitter release (Korolchuk et al., 2007; Inoshita et al., 2017). In clones of fly photoreceptors, loss of either *Vps26* or *Vps35* affects the recycling of Rhodopsin upon light exposure, a dramatic increase in late endosomes and lysosomes, and ultimately the degeneration of photoreceptors (Wang et al., 2014). A significant increase in GlcCer in photoreceptors was also observed in mutant *Vps26* and *Vps35* photoreceptors (Lin et al., 2018), indicating a role of GlcCer in the neurodegeneration caused by retromer dysfunction. In contrast, loss of *Vps29* does not cause developmental lethality. However, *Vps29* mutants phenocopy the NMJ defects of *Vps35* mutant larvae and have an activity-dependent neurodegeneration in adult photoreceptors (Wang et al., 2014; Inoshita et al., 2017; Ye et al., 2020). In addition, *Vps29* mutant flies exhibit shortened lifespan and age-dependent locomotor defects (Ye et al., 2020).

Loss of *Vps35* also impairs α-Syn degradation and exacerbates its neurotoxicity in flies (Miura et al., 2014). This increase in α-Syn toxicity was attributed to a disruption in trafficking of lysosomal proteases (Miura et al., 2014). However, as mentioned, an accumulation of Cer/GlcCer was observed in *Vps35* mutant clones, suggesting that SL dysmetabolism may also be involved (Lin et al., 2018). If GlcCer facilitates the aggregation of α-Syn, the *Vps35* deficiency may promote its toxicity. Note that *Vps35*- and *Gba1b*-associated neurodegeneration in photoreceptors are both activity dependent, supporting a role for GlcCer-mediated α-Syn neurotoxicity when *Vps35* is lost or reduced (Wang et al., 2014, 2022c).

### PLA2G6

Biallelic variants of *PLA2G6* cause three neurological disorders: early adulthood-onset dystonia-parkinsonism (PARK14, OMIM# 612953), early childhood to juvenile-onset atypical neuroaxonal dystrophy (OMIM# 610217), and infantile neuroaxonal dystrophy (INAD, OMIM# 256600). *PLA2G6* encodes a phospholipase. Interestingly, a fly model of *PLA2G6* deficiency uncovered that neuronal accumulation of Cer/GlcCer is a major contributor to the development of the disease (Lin et al., 2018). The fly ortholog of *PLA2G6* is *iPLA2-VIA*. Loss of *iPLA2-VIA* in flies causes a short lifespan and neurodegeneration. Lipidomics studies revealed that the levels of many SL species, but not phospholipids, are elevated in the *iPLA2-VIA* mutant flies. Biochemical assays showed that iPLA2-VIA binds to Vps26 and Vps35, independent of its phospholipase activity and that an enzyme-dead protein is able to rescue the mutant phenotypes (Lin et al., 2018). iPLA2-VIA deficiency causes a significant reduction of both Vps26 and Vps35 and an impairment of retromer function, leading to an imbalance in the routing of endosomal components, including Cer/GlcCer, to lysosomes. Hence, lysosomal trafficking is disrupted and Cer/GlcCer accumulates in lysosomes, both of which lead to lysosome expansion and dysfunction, ultimately causing the demise of neurons. In attempt to rescue phenotypes, Cer synthesis was suppressed in three ways: knocking down fly SPT subunit *lace*, treatment with SPT inhibitor myriocin, and with the sphingomyelinase inhibitor, desipramine. In all three ways the cellular and the neurological defects of the mutant flies are rescued, showing that Cer accumulation accounts for many of the neurological defects caused by *iPLA2-VIA* deficiency (Lin et al., 2018).

In a follow up study, Cer accumulation and lysosomal expansion phenotypes were observed in INAD patient cells. Moreover, an accumulation of Cer in Purkinje cells and DA neurons was also observed in *PLA2G6* mutant mice, arguing that the neuropathological mechanisms are evolutionary conserved. Drugs targeting the endolysosomal pathway suppress some of the phenotypes and alleviate lysosomal stress in human cells and in flies (Lin et al., 2023).

### PINK1

Variants in *PINK1* cause an AR, early onset form of parkinsonism (PARK6, OMIM# 605909). *PINK1* encodes a serine/threonine kinase PINK1, which localizes to mitochondria and performs important roles in mitochondrial homeostasis together with Parkin, another PD risk factor encoded by the *PRKN* gene (McWilliams and Muqit, 2017). In contrast to mice models for *Pink1*, which do not exhibit gross physiological, neurological or behavioral phenotype (Paul and Pickrell, 2021), fly *Pink1* mutants exhibit obvious phenotypes, including shortened lifespan and early onset locomotor defects. Mutant flies also exhibit mitochondrial dysfunction in muscles and DA neurons, which leads to the degeneration of myocytes and DA neurons in aged flies (Clark et al., 2006; Park et al., 2006). Similar phenotypic and pathological changes were observed in *Pink1* knockdown fly models (Wang et al., 2006; Yang et al., 2006). Loss of *Pink1* affects mitochondrial homeostasis by modulating the mitochondrial fission/fusion machinery and mitophagy, a selective autophagic process targeting damaged or dysfunctional mitochondria (Deng et al., 2008; Poole et al., 2008; Yang et al., 2008; Cornelissen et al., 2018; Kim et al., 2019). A recent study uncovered that the *Pink1*-associated mitochondrial defects are, at least partially, mediated by Cer accumulation (Vos et al., 2021). Increased Cer levels were also observed in *Pink1*^–/–^ mouse embryonic fibroblasts, *Pink1* mutant fly muscles and *PINK1*-PD patient fibroblasts. The defects in ATP levels and mitochondrial morphology in *Pink1* mutant flies were effectively rescued by either knocking down fly ceramide synthase *schlank* or by treating flies with the SPT inhibitor myriocin, again providing evidence for a role for Cer accumulation in the pathogenesis of the disease. Further, Cer accumulation induces mitophagy, which the authors proposed facilitates the clearance of damaged mitochondria in *Pink1* mutant animals (Vos et al., 2021). How *Pink1* loss induces changes in Cer homeostasis remains to be determined.

## Future directions

### Which SL species are toxic?

Sphingolipids correspond to a large collection of lipids, and an obvious question is: which SL species accumulate and facilitate neurotoxicity? In the diseases discussed herein, an accumulation of Cer/GlcCer is most commonly observed. However, in no case was an accumulation of only one SL species observed. In the INAD fly model, loss of *iPLA2-VIA* causes elevated level of Sph, Cer and GlcCer. The mutant phenotypes are alleviated by suppression of either *de novo* synthesis or SM hydrolysis, suggesting that Cers may be the toxic SL species. However, inhibiting GlcCer synthesis with Miglustat does not improve the phenotypes, suggesting that GlcCer is not a major contributor to pathogenesis (Lin et al., 2023). However, in GD GlcCer is the major SL that accumulates in cells and in the *Gba1b* mutant fly model a 16-fold increase of GlcCer is observed in fly heads (Kinghorn et al., 2016; Wang et al., 2022c). This indicates that the GlcCer induced toxicity is due to its very elevated level. In the *SPTLC1*-ALS and *SPTSSA*-HSP cases, elevated *de novo* synthesis induces increased levels of dhSph, Cer as well as downstream products such as GlcCer and SM (Johnson et al., 2021; Mohassel et al., 2021; Srivastava et al., 2023). In summary, the complexity of SL metabolism makes it difficult to determine which SL species cause toxicity. However, simultaneously lowering many SLs by suppressing *de novo* synthesis suppress phenotypes in several fly models, including INAD, FRDA, and *PINK1*-PD, suggesting that suppressing *de novo* synthesis may be a viable therapeutic strategy for many diseases (Chen et al., 2016b; Lin et al., 2018; Vos et al., 2021).

### How to accurately detect SL accumulation?

Lipids are commonly detected using mass spectrometry-dependent approaches. However, the extreme structural diversity of SLs prevents the measurement of all SLs, or even all Cers, in a cost-effective manner (Pruett et al., 2008). Discriminating between GlcCer and GalCer by mass spectrometry requires additional efforts (Boutin et al., 2016). Hence, the two species are often labeled collectively as hexosylceramide (HexCer). Currently, the common choice for measuring SL is either a targeted approach for a specific SL subclass (Cer as the most common choice) or an unbiased large-scale lipidomic approach that encompasses both SLs and other lipids. An unbiased “sphingolipidomic” assay should be developed to facilitate a time- and cost-efficient way of measuring SLs.

The choice of sample types is also key to provide a precise landscape of SL changes in diseases. Limited by sample accessibility, blood cells and plasma/serum are the most commonly used samples. Most SLs including Cers are incorporated in membranes in cells and are not soluble in an aqueous environment such as cytosol and plasma. SLs are transported to the plasma membrane, extruded, and captured by lipoproteins in plasma (Iqbal et al., 2017). When cells become dysfunctional, SL species may not be efficiently transported to the plasma membrane, which may affect their distribution in lipoproteins (Wang et al., 2018). Hence, SL levels in plasma may not reflect what is happening in neurons and glia. In apolipoprotein bound SL in plasma, SM is the dominant species (∼87%) while Cer and HexCer correspond to only ∼6% (Hammad et al., 2010). Some metabolic disorders also affect plasma SL levels, which may mask the plasma SL profile changes caused by the SL dysmetabolism in the nervous system. For example, increased levels of multiple SLs including SM, Cer and GSLs are observed in individuals with diabetes (Russo et al., 2013). For some neurological diseases, blood cells or fibroblasts may reflect the status of SL metabolism in the nervous system better than what is observed in plasma. For example, elevated levels of Cers are observed in FRDA patient skin fibroblasts (Wang et al., 2022b), consistent with the observations in *fh* mutant flies and patient heart samples (Chen et al., 2016a,b). However, in patient plasma samples levels of Cers with C16-C18 acyl chain do not significantly change, while levels of VLCFA-Cers decrease (Wang et al., 2022a). Given that sampling neurons of patients is challenging, neuronal cells derived from induced pluripotent stem cells (iPSCs) or transdifferentiated from blood cells or fibroblasts can be used as surrogates and may provide the best source for SL profiling (Engle et al., 2018; Mollinari et al., 2018). In sum, a systematic lipidomics-based analysis of various cell types (blood cells, fibroblasts and induced neurons) and body fluids (serum and CSF) may provide a more precise read-out of SL changes in the nervous system.

### How are SL levels altered?

The accumulation of SLs such as Cer could be a consequence of either increased synthesis or decreased degradation. The *SPTLC1*- and *SPTSSA*-associated disorders represent the former case, while Farber Disease (deficiency of ceramidase encoded by *ASAH1*) corresponds to an example of the latter case. Increased anabolism as well as reduced catabolism can also occur in the same disease. For example, in the case of fly INAD model, the disruption of retromer function causes an increase in endolysosomal trafficking and an accumulation of SLs. However, through an unknown process, the *de novo* synthesis pathway is also activated, given that the dhSph level is elevated. Moreover, inhibition of *de novo* synthesis by *lace* knockdown or by myriocin treatment, or knockdown of the salvage pathway using the SM inhibitor, desipramine, both effectively rescue the defects in the disease model, showing that both pathways contributed to disease progression (Lin et al., 2018).

The source of SL accumulation may not be obvious when the disease is not directly caused by defects in anabolism or catabolism of SLs, as for example in FRDA and *PINK1*-PD. In models of both of these diseases, iron accumulation and mitochondrial defects are observed (Esposito et al., 2013; Chen et al., 2016b). How these changes lead to Cer accumulation and whether the two diseases share a similar mechanism is not yet established.

### Bridging the defects: Mitochondria and lysosomes

Mitochondria and lysosome are the two most important organelles that are often affected in PD (Haelterman et al., 2014; Vazquez-Velez and Zoghbi, 2021; Ye et al., 2022). The driver of the disease pathogenesis may be either one or both of these organelles. For example, variants in the *PINK1* and *PRKN* genes mainly cause mitochondrial dysfunction, while *VPS35* and *PLA2G6* deficiency mainly result in lysosomal deficits. However, both organelles seem to be affected as the diseases progress. Similar inter-organelle influences can also be observed in rare NDDs when the defects seem to originate from one organelle, such as in LSDs (Stepien et al., 2020).

In NDDs that primarily affect mitochondria or lysosomes, an accumulation of SLs (especially Cers) has been observed. Hence, Cer accumulation may bridge the defects in both organelles and underlie a synergy between the two sources to promote neurodegeneration. Increased levels of Cers in membranous structures stiffen membranes and impair vesicular trafficking (Huttner and Zimmerberg, 2001; Castro et al., 2014). Hence, an increase in Cer levels caused by mitochondrial dysfunction, such as that in *Pink1*/*PINK1* models (Vos et al., 2021), may disrupt endolysosomal trafficking and cause lysosomal defects. In contrast, an increased production of Cer in lysosomes may also disrupt mitochondrial homeostasis, as observed in the *PLA2G6* models (Lin et al., 2018, 2023).

### Origin of the defects: Neurons versus glia

The cellular origin of the defects that cause NDDs is not always obvious. Studies in flies have revealed that defects in SL metabolism originate in glial cells when *dACOX1* or *Gba1b* is lost (Chung et al., 2022; Wang et al., 2022c). Expression of both *dACOX1* and *Gba1b* are highly enriched in glial cells. In the *dACOX1* model, loss of *dACOX1* causes increased levels of VLCFA-Cer and S1P in glial cells, while the glia-derived S1P is transferred to neurons where it induces neurotoxicity (Chung et al., 2022). In the *Gba1b* case, GlcCer is produced in neurons. However, it is transferred to glia where it accumulates, causing glia to be affected before neurons (Wang et al., 2022c).

In contrast to *Gba1b*, *iPLA2-VIA* is mainly expressed in neurons. In both *Gba1b* and *iPLA2-VIA* mutants an elevation of Cer/GlcCer is observed. However, the accumulation of GlcCer occurs only in neurons when *iPLA2-VIA* is lost, whereas GlcCer accumulation occurs first in glia, then in neurons, when *Gba1b* is lost (Lin et al., 2018; Wang et al., 2022c). Interestingly, both *PLA2G6* and *GBA* genes are associated with PD/parkinsonism and the fly models share many similar phenotypes (Lin et al., 2018; Wang et al., 2022c) yet the data clearly show that the cellular origin of the phenotypes is quite different.

### Sphingolipids as potential drug targets

The idea of modulating cellular SL levels is not novel. Miglustat, an inhibitor of ceramide glucosyltransferase, has been approved by the FDA to be used to treat type I GD. Treatment with Miglustat effectively alleviates the symptoms in type I GD patients such as hepatosplenomegaly and anemia (Ficicioglu, 2008). However, a clinical trial with Miglustat failed to show an improvement in neurological defects in type III GD patients (Schiffmann et al., 2008). In addition to GD, Miglustat has been shown to provide beneficial effects in NPC (Patterson et al., 2007) and Sandhoff Disease (OMIM# 268800) (Tallaksen and Berg, 2009; Masciullo et al., 2010). In both diseases, accumulation of GSLs derived from GlcCer is observed (Newton et al., 2018; Breiden and Sandhoff, 2019). However, due to its specificity to downregulate GlcCer synthesis and its derivatives, Miglustat was not tested in other types of sphingolipidosis, such as Krabbe Disease (GalCer) and Niemann-Pick A/B (SM).

The disease-causing variants in *SPTLC1* and *SPTSSA* lead to an elevation of the *de novo* synthesis (Johnson et al., 2021; Mohassel et al., 2021; Srivastava et al., 2023), suggesting that inhibiting the SPT complex should suppress the associated phenotypes. Suppression of the *de novo* synthesis should lower levels of many, if not most, of the downstream SL species. SPT inhibition, either genetically or pharmacologically, has been shown to alleviate the neurological phenotypes in disease models for FRDA, INAD, and *PINK1*-PD, suggesting a strategy for treatment (Chen et al., 2016b; Lin et al., 2018; Vos et al., 2021). In model organisms, myriocin is a commonly used SPT inhibitor. However, oral administration of myriocin has been shown to cause strong intestinal toxicity and the ability of myriocin to cross the BBB is not established (Osuchowski et al., 2004). Hence, novel SPT inhibitors, which have low toxicity and the ability to cross the BBB, are desirable.

## Author contributions

HB and XP contributed to the conception of the review. XP wrote the first draft of the manuscript. XP, DD, SL, and HB wrote sections of the manuscript. All authors contributed to manuscript revision, read, and approved the submitted version.

## References

[B1] Aaltonen M. J. Alecu I. Konig T. Bennett S. A. Shoubridge E. A. (2022). Serine palmitoyltransferase assembles at ER-mitochondria contact sites. *Life Sci. Alliance* 5:e202101278. 10.26508/lsa.202101278 34785538 PMC8605320

[B2] Adachi-Yamada T. Gotoh T. Sugimura I. Tateno M. Nishida Y. Onuki T. (1999). De novo synthesis of sphingolipids is required for cell survival by down-regulating c-Jun N-terminal kinase in *Drosophila* imaginal discs. *Mol. Cell Biol.* 19 7276–7286. 10.1128/MCB.19.10.7276 10490662 PMC84720

[B3] Agrawal S. Tuthill J. C. (2022). The two-body problem: Proprioception and motor control across the metamorphic divide. *Curr. Opin. Neurobiol.* 74:102546. 10.1016/j.conb.2022.102546 35512562 PMC9167723

[B4] Albert J. T. Gopfert M. C. (2015). Hearing in *Drosophila*. *Curr. Opin. Neurobiol.* 34 79–85. 10.1016/j.conb.2015.02.001 25710304 PMC4582067

[B5] Ali Y. O. Escala W. Ruan K. Zhai R. G. (2011). Assaying locomotor, learning, and memory deficits in *Drosophila* models of neurodegeneration. *J. Vis. Exp.* 49:2504. 10.3791/2504 21445036 PMC3197301

[B6] Amberger J. S. Bocchini C. A. Scott A. F. Hamosh A. (2019). OMIM.org: leveraging knowledge across phenotype-gene relationships. *Nucleic Acids Res.* 47 D1038–D1043. 10.1093/nar/gky1151 30445645 PMC6323937

[B7] Anderson P. R. Kirby K. Hilliker A. J. Phillips J. P. (2005). RNAi-mediated suppression of the mitochondrial iron chaperone, frataxin, in *Drosophila*. *Hum. Mol. Genet.* 14 3397–3405. 10.1093/hmg/ddi367 16203742

[B8] Area-Gomez E. Larrea D. Yun T. Xu Y. Hupf J. Zandkarimi F. (2021). Lipidomics study of plasma from patients suggest that ALS and PLS are part of a continuum of motor neuron disorders. *Sci. Rep.* 11:13562. 10.1038/s41598-021-92112-3 34193885 PMC8245424

[B9] Armstrong M. J. Okun M. S. (2020). Diagnosis and treatment of parkinson disease: a review. *JAMA* 323 548–560. 10.1001/jama.2019.22360 32044947

[B10] Aryal B. Lee Y. (2019). Disease model organism for Parkinson disease: *Drosophila* melanogaster. *BMB Rep.* 52 250–258. 10.5483/BMBRep.2019.52.4.204 30545438 PMC6507844

[B11] Atkinson D. Nikodinovic Glumac J. Asselbergh B. Ermanoska B. Blocquel D. Steiner R. (2017). Sphingosine 1-phosphate lyase deficiency causes charcot-marie-tooth neuropathy. *Neurology* 88 533–542. 10.1212/WNL.0000000000003595 28077491 PMC5304460

[B12] Auer T. O. Benton R. (2016). Sexual circuitry in *Drosophila*. *Curr. Opin. Neurobiol.* 38 18–26. 10.1016/j.conb.2016.01.004 26851712

[B13] Auluck P. K. Chan H. Y. Trojanowski J. Q. Lee V. M. Bonini N. M. (2002). Chaperone suppression of alpha-synuclein toxicity in a *Drosophila* model for Parkinson’s disease. *Science* 295 865–868. 10.1126/science.1067389 11823645

[B14] Azuma N. O’Brien J. S. Moser H. W. Kishimoto Y. (1994). Stimulation of acid ceramidase activity by saposin D. *Arch. Biochem. Biophys.* 311 354–357. 10.1006/abbi.1994.1248 8203897

[B15] Azuma Y. Mizuta I. Tokuda T. Mizuno T. (2018). Amyotrophic lateral sclerosis model. *Adv. Exp. Med. Biol.* 1076 79–95. 10.1007/978-981-13-0529-0_6 29951816

[B16] Bandres-Ciga S. Saez-Atienzar S. Kim J. J. Makarious M. B. Faghri F. Diez-Fairen M. (2020). Large-scale pathway specific polygenic risk and transcriptomic community network analysis identifies novel functional pathways in Parkinson disease. *Acta Neuropathol.* 140 341–358. 10.1007/s00401-020-02181-3 32601912 PMC8096770

[B17] Barbagallo B. Garrity P. A. (2015). Temperature sensation in *Drosophila*. *Curr. Opin. Neurobiol.* 34 8–13. 10.1016/j.conb.2015.01.002 25616212 PMC4508239

[B18] Bauer R. Voelzmann A. Breiden B. Schepers U. Farwanah H. Hahn I. (2009). Schlank, a member of the ceramide synthase family controls growth and body fat in *Drosophila*. *EMBO J.* 28 3706–3716. 10.1038/emboj.2009.305 19834458 PMC2790492

[B19] Bellen H. J. Tong C. Tsuda H. (2010). 100 years of *Drosophila* research and its impact on vertebrate neuroscience: a history lesson for the future. *Nat. Rev. Neurosci.* 11 514–522. 10.1038/nrn2839 20383202 PMC4022039

[B20] Bellen H. J. Wangler M. F. Yamamoto S. (2019). The fruit fly at the interface of diagnosis and pathogenic mechanisms of rare and common human diseases. *Hum. Mol. Genet.* 28 R207–R214. 10.1093/hmg/ddz135 31227826 PMC6872428

[B21] Bickert A. Ginkel C. Kol M. vom Dorp K. Jastrow H. Degen J. (2015). Functional characterization of enzymes catalyzing ceramide phosphoethanolamine biosynthesis in mice. *J. Lipid Res.* 56 821–835. 10.1194/jlr.M055269 25667419 PMC4373740

[B22] Biegstraaten M. van Schaik I. N. Aerts J. M. Hollak C. E. (2008). ‘Non-neuronopathic’ Gaucher disease reconsidered. Prevalence of neurological manifestations in a dutch cohort of type I gaucher disease patients and a systematic review of the literature. *J. Inherit. Metab. Dis.* 31 337–349. 10.1007/s10545-008-0832-y 18404411

[B23] Blasco H. Veyrat-Durebex C. Bocca C. Patin F. Vourc’h P. Kouassi Nzoughet J. (2017). Lipidomics reveals cerebrospinal-fluid signatures of ALS. *Sci. Rep.* 7:17652. 10.1038/s41598-017-17389-9 29247199 PMC5732162

[B24] Blauwendraat C. Nalls M. A. Singleton A. B. (2020a). The genetic architecture of Parkinson’s disease. *Lancet Neurol.* 19 170–178. 10.1016/S1474-4422(19)30287-X 31521533 PMC8972299

[B25] Blauwendraat C. Reed X. Krohn L. Heilbron K. Bandres-Ciga S. Tan M. (2020b). Genetic modifiers of risk and age at onset in GBA associated Parkinson’s disease and lewy body dementia. *Brain* 143 234–248. 10.1093/brain/awz350 31755958 PMC6935749

[B26] Bode H. Bourquin F. Suriyanarayanan S. Wei Y. Alecu I. Othman A. (2016). HSAN1 mutations in serine palmitoyltransferase reveal a close structure-function-phenotype relationship. *Hum. Mo.l Genet.* 25 853–865. 10.1093/hmg/ddv611 26681808

[B27] Boutin M. Sun Y. Shacka J. J. Auray-Blais C. (2016). Tandem mass spectrometry multiplex analysis of glucosylceramide and galactosylceramide isoforms in brain tissues at different stages of Parkinson disease. *Anal. Chem.* 88 1856–1863. 10.1021/acs.analchem.5b04227 26735924

[B28] Bradova V. Smid F. Ulrich-Bott B. Roggendorf W. Paton B. C. Harzer K. (1993). Prosaposin deficiency: further characterization of the sphingolipid activator protein-deficient sibs. Multiple glycolipid elevations (including lactosylceramidosis), partial enzyme deficiencies and ultrastructure of the skin in this generalized sphingolipid storage disease. *Hum. Genet,.* 92 143–152. 10.1007/BF00219682 8370580

[B29] Breiden B. Sandhoff K. (2019). Lysosomal glycosphingolipid storage diseases. *Annu. Rev. Biochem.* 88 461–485. 10.1146/annurev-biochem-013118-111518 31220974

[B30] Breiden B. Sandhoff K. (2021). Acid sphingomyelinase, a lysosomal and secretory phospholipase C, is key for cellular phospholipid catabolism. *Int. J. Mol. Sci.* 22:9001. 10.3390/ijms22169001 34445706 PMC8396676

[B31] Bultron G. Kacena K. Pearson D. Boxer M. Yang R. Sathe S. (2010). The risk of Parkinson’s disease in type 1 Gaucher disease. *J. Inherit. Metab. Dis.* 33 167–173. 10.1007/s10545-010-9055-0 20177787 PMC2887303

[B32] Campuzano V. Montermini L. Lutz Y. Cova L. Hindelang C. Jiralerspong S. (1997). Frataxin is reduced in Friedreich ataxia patients and is associated with mitochondrial membranes. *Hum. Mol. Genet.* 6 1771–1780. 10.1093/hmg/6.11.1771 9302253

[B33] Capablo J. L. Saenz de Cabezon A. Fraile J. Alfonso P. Pocovi M. Giraldo P. (2008). Neurological evaluation of patients with Gaucher disease diagnosed as type 1. *J. Neurol. Neurosurg. Psychiatry* 79 219–222. 10.1136/jnnp.2006.111518 17682016

[B34] Castillo S. S. Teegarden D. (2003). Sphingosine-1-phosphate inhibition of apoptosis requires mitogen-activated protein kinase phosphatase-1 in mouse fibroblast C3H10T 1/2 cells. *J. Nutr.* 133 3343–3349. 10.1093/jn/133.11.3343 14608042

[B35] Castro B. M. Prieto M. Silva L. C. (2014). Ceramide: a simple sphingolipid with unique biophysical properties. *Prog. Lipid Res.* 54 53–67. 10.1016/j.plipres.2014.01.004 24513486

[B36] Chao Y. X. Lee B. Ng E. Y. Lian M. M. Chew E. G. Y. Tandiono M. (2021). Association analysis of PSAP variants in Parkinson’s disease patients. *Brain* 144:e9. 10.1093/brain/awaa358 33221828

[B37] Chen K. Ho T. S. Lin G. Tan K. L. Rasband M. N. Bellen H. J. (2016a). Loss of Frataxin activates the iron/sphingolipid/PDK1/Mef2 pathway in mammals. *eLife* 5:e20732. 10.7554/eLife.20732 27901468 PMC5130293

[B38] Chen K. Lin G. Haelterman N. A. Ho T. S. Li T. Li Z. (2016b). Loss of frataxin induces iron toxicity, sphingolipid synthesis, and Pdk1/Mef2 activation, leading to neurodegeneration. *eLife* 5:e16043. 10.7554/eLife.16043 27343351 PMC4956409

[B39] Chen X. Li J. Gao Z. Yang Y. Kuang W. Dong Y. (2022). Endogenous ceramide phosphoethanolamine modulates circadian rhythm via neural-glial coupling in *Drosophila*. *Nat. Sci. Rev.* 9:nwac148. 10.1093/nsr/nwac148 36713590 PMC9875363

[B40] Chouhan A. K. Guo C. Hsieh Y. C. Ye H. Senturk M. Zuo Z. (2016). Uncoupling neuronal death and dysfunction in *Drosophila* models of neurodegenerative disease. *Acta Neuropathol. Commun.* 4:62. 10.1186/s40478-016-0333-4 27338814 PMC4918017

[B41] Chung H. L. Wangler M. F. Marcogliese P. C. Jo J. Ravenscroft T. A. Zuo Z. (2020). Loss- or gain-of-function mutations in ACOX1 cause axonal loss via different mechanisms. *Neuron* 106 589.e6–606.e6. 10.1016/j.neuron.2020.02.021 32169171 PMC7289150

[B42] Chung H. Ye Q. Park Y.-J. Zuo Z. Kanca O. Mok J.-W. (2022). Very long-chain fatty acids induce glial-derived sphingosine-1-phosphate synthesis, secretion, and neuroinflammation. *SSRN* [preprint]. 10.2139/ssrn.4121836 PMC10160010 37084732

[B43] Clark I. E. Dodson M. W. Jiang C. Cao J. H. Huh J. R. Seol J. H. (2006). *Drosophila* pink1 is required for mitochondrial function and interacts genetically with parkin. *Nature* 441 1162–1166. 10.1038/nature04779 16672981

[B44] Clark M. Q. Zarin A. A. Carreira-Rosario A. Doe C. Q. (2018). Neural circuits driving larval locomotion in *Drosophila*. *Neural. Dev.* 13:6. 10.1186/s13064-018-0103-z 29673388 PMC5907184

[B45] Cognigni P. Felsenberg J. Waddell S. (2018). Do the right thing: Neural network mechanisms of memory formation, expression and update in *Drosophila*. *Curr. Opin. Neurobiol.* 49 51–58. 10.1016/j.conb.2017.12.002 29258011 PMC5981003

[B46] Cook A. Giunti P. (2017). Friedreich’s ataxia: clinical features, pathogenesis and management. *Br. Med. Bull.* 124 19–30. 10.1093/bmb/ldx034 29053830 PMC5862303

[B47] Cornelissen T. Vilain S. Vints K. Gounko N. Verstreken P. Vandenberghe W. (2018). Deficiency of parkin and PINK1 impairs age-dependent mitophagy in *Drosophila*. *eLife* 7:e35878. 10.7554/eLife.35878 29809156 PMC6008047

[B48] Custodia A. Aramburu-Nunez M. Correa-Paz C. Posado-Fernandez A. Gomez-Larrauri A. Castillo J. (2021). Ceramide metabolism and Parkinson’s disease-therapeutic targets. *Biomolecules* 11:945. 10.3390/biom11070945 34202192 PMC8301871

[B49] Cutler R. G. Pedersen W. A. Camandola S. Rothstein J. D. Mattson M. P. (2002). Evidence that accumulation of ceramides and cholesterol esters mediates oxidative stress-induced death of motor neurons in amyotrophic lateral sclerosis. *Ann. Neurol.* 52 448–457. 10.1002/ana.10312 12325074

[B50] Cuvillier O. Pirianov G. Kleuser B. Vanek P. G. Coso O. A. Gutkind S. (1996). Suppression of ceramide-mediated programmed cell death by sphingosine-1-phosphate. *Nature* 381 800–803. 10.1038/381800a0 8657285

[B51] D’Angelo G. Capasso S. Sticco L. Russo D. (2013). Glycosphingolipids: synthesis and functions. *FEBS J.* 280 6338–6353. 10.1111/febs.12559 24165035

[B52] Danzer K. M. Kranich L. R. Ruf W. P. Cagsal-Getkin O. Winslow A. R. Zhu L. (2012). Exosomal cell-to-cell transmission of alpha synuclein oligomers. *Mol. Neurodegener* 7:42. 10.1186/1750-1326-7-42 22920859 PMC3483256

[B53] Dasgupta U. Bamba T. Chiantia S. Karim P. Tayoun A. N. Yonamine I. (2009). Ceramide kinase regulates phospholipase C and phosphatidylinositol 4, 5, bisphosphate in phototransduction. *Proc. Natl. Acad. Sci. U.S.A.* 106 20063–20068. 10.1073/pnas.0911028106 19892737 PMC2785292

[B54] Davis D. L. Gable K. Suemitsu J. Dunn T. M. Wattenberg B. W. (2019). The ORMDL/Orm-serine palmitoyltransferase (SPT) complex is directly regulated by ceramide: reconstitution of SPT regulation in isolated membranes. *J. Biol. Chem.* 294 5146–5156. 10.1074/jbc.RA118.007291 30700557 PMC6442065

[B55] Davis M. Y. Trinh K. Thomas R. E. Yu S. Germanos A. A. Whitley B. N. (2016). Glucocerebrosidase deficiency in *Drosophila* results in alpha-synuclein-independent protein aggregation and neurodegeneration. *PLoS Genet.* 12:e1005944. 10.1371/journal.pgen.1005944 27019408 PMC4809718

[B56] Dawkins J. L. Hulme D. J. Brahmbhatt S. B. Auer-Grumbach M. Nicholson G. A. (2001). Mutations in SPTLC1, encoding serine palmitoyltransferase, long chain base subunit-1, cause hereditary sensory neuropathy type I. *Nat. Genet.* 27 309–312. 10.1038/85879 11242114

[B57] de Andres-Bragado L. Sprecher S. G. (2019). Mechanisms of vision in the fruit fly. *Curr. Opin. Insect. Sci.* 36, 25–32. 10.1016/j.cois.2019.06.005 31325739

[B58] de Lau L. M. Breteler M. M. (2006). Epidemiology of Parkinson’s disease. *Lancet Neurol.* 5 525–535. 10.1016/S1474-4422(06)70471-9 16713924

[B59] DeAngelis B. D. Zavatone-Veth J. A. Clark D. A. (2019). The manifold structure of limb coordination in walking *Drosophila*. *Elife* 8:e46409. 10.7554/eLife.46409 31250807 PMC6598772

[B60] Deng B. Li Q. Liu X. Cao Y. Li B. Qian Y. (2019). Chemoconnectomics: Mapping chemical transmission in *Drosophila*. *Neuron* 101 876–893.e4. 10.1016/j.neuron.2019.01.045 30799021

[B61] Deng H. Dodson M. W. Huang H. Guo M. (2008). The Parkinson’s disease genes pink1 and parkin promote mitochondrial fission and/or inhibit fusion in *Drosophila*. *Proc. Natl. Acad. Sci. U.S.A.* 105 14503–14508. 10.1073/pnas.0803998105 18799731 PMC2567186

[B62] Deng H. Yuan L. (2014). Genetic variants and animal models in SNCA and Parkinson disease. *Ageing Res. Rev.* 15 161–176. 10.1016/j.arr.2014.04.002 24768741

[B63] Diao F. Ironfield H. Luan H. Diao F. Shropshire W. C. Ewer J. (2015). Plug-and-play genetic access to *drosophila* cell types using exchangeable exon cassettes. *Cell Rep.* 10 1410–1421. 10.1016/j.celrep.2015.01.059 25732830 PMC4373654

[B64] Dodge J. C. Treleaven C. M. Pacheco J. Cooper S. Bao C. Abraham M. (2015). Glycosphingolipids are modulators of disease pathogenesis in amyotrophic lateral sclerosis. *Proc. Natl. Acad. Sci. U.S.A.* 112 8100–8105. 10.1073/pnas.1508767112 26056266 PMC4491749

[B65] Dung V. M. Thao D. T. P. (2018). Parkinson’s disease model. *Adv. Exp. Med. Biol.* 1076 41–61. 10.1007/978-981-13-0529-0_4 29951814

[B66] Engle S. J. Blaha L. Kleiman R. J. (2018). Best practices for translational disease modeling using human iPSC-derived neurons. *Neuron* 100 783–797. 10.1016/j.neuron.2018.10.033 30465765

[B67] Esfandiary A. Finkelstein D. I. Voelcker N. H. Rudd D. (2022). Clinical sphingolipids pathway in parkinson’s disease: from GCase to integrated-biomarker discovery. *Cells* 11:1353. 10.3390/cells11081353 35456032 PMC9028315

[B68] Esposito G. Vos M. Vilain S. Swerts J. De Sousa Valadas J. Van Meensel S. (2013). Aconitase causes iron toxicity in *Drosophila* pink1 mutants. *PLoS Genet.* 9:e1003478. 10.1371/journal.pgen.1003478 23637640 PMC3636082

[B69] Facchi D. Rimoldi V. Straniero L. Paraboschi E. M. Solda G. Zecchinelli A. L. (2020). Saposin D variants are not a common cause of familial Parkinson’s disease among Italians. *Brain* 143:e71. 10.1093/brain/awaa213 32793950

[B70] Feany M. B. Bender W. W. (2000). A *Drosophila* model of Parkinson’s disease. *Nature* 404 394–398. 10.1038/35006074 10746727

[B71] Fearnley J. M. Lees A. J. (1991). Ageing and Parkinson’s disease: substantia nigra regional selectivity. *Brain* 114(Pt 5), 2283–2301. 10.1093/brain/114.5.2283 1933245

[B72] Ferdinandusse S. Denis S. Hogenhout E. M. Koster J. van Roermund C. W. IJlst L. (2007). Clinical, biochemical, and mutational spectrum of peroxisomal acyl-coenzyme A oxidase deficiency. *Hum. Mutat.* 28 904–912. 10.1002/humu.20535 17458872

[B73] FernAndez-Eulate G. Ruiz-Sanz J. I. Riancho J. ZufirIa M. GereNu G. FernAndez-TorrOn R. (2020). A comprehensive serum lipidome profiling of amyotrophic lateral sclerosis. *Amyotroph Lateral Scler. Frontotemporal Degener.* 21 252–262. 10.1080/21678421.2020.1730904 32106710

[B74] Ficicioglu C. (2008). Review of miglustat for clinical management in gaucher disease type 1. *Ther. Clin. Risk Manag.* 4 425–431. 10.2147/tcrm.s6865 18728838 PMC2504062

[B75] Fields C. R. Bengoa-Vergniory N. Wade-Martins R. (2019). Targeting alpha-synuclein as a therapy for Parkinson’s disease. *Front. Mol. Neurosci.* 12:299. 10.3389/fnmol.2019.00299 31866823 PMC6906193

[B76] Fleming S. M. Davis A. Simons E. (2022). Targeting alpha-synuclein via the immune system in Parkinson’s disease: current vaccine therapies. *Neuropharmacology* 202:108870. 10.1016/j.neuropharm.2021.108870 34742741 PMC10251253

[B77] Follett J. Norwood S. J. Hamilton N. A. Mohan M. Kovtun O. Tay S. (2014). The Vps35 D620N mutation linked to Parkinson’s disease disrupts the cargo sorting function of retromer. *Traffic* 15 230–244. 10.1111/tra.12136 24152121

[B78] Fortuna F. Barboni P. Liguori R. Valentino M. L. Savini G. Gellera C. (2009). Visual system involvement in patients with Friedreich’s ataxia. *Brain* 132(Pt 1), 116–123. 10.1093/brain/awn269 18931386

[B79] Fournier B. Saudubray J. M. Benichou B. Lyonnet S. Munnich A. Clevers H. (1994). Large deletion of the peroxisomal acyl-CoA oxidase gene in pseudoneonatal adrenoleukodystrophy. *J. Clin. Invest.* 94 526–531. 10.1172/JCI117365 8040306 PMC296126

[B80] Freeman M. R. Doherty J. (2006). Glial cell biology in *Drosophila* and vertebrates. *Trends Neurosci.* 29 82–90. 10.1016/j.tins.2005.12.002 16377000

[B81] Fridman V. Suriyanarayanan S. Novak P. David W. Macklin E. A. McKenna-Yasek D. (2019). Randomized trial of l-serine in patients with hereditary sensory and autonomic neuropathy type 1. *Neurology* 92 e359–e370. 10.1212/WNL.0000000000006811 30626650 PMC6345118

[B82] Frye M. A. (2010). Multisensory systems integration for high-performance motor control in flies. *Curr. Opin. Neurobiol.* 20 347–352. 10.1016/j.conb.2010.02.002 20202821 PMC3635923

[B83] Fujita N. Suzuki K. Vanier M. T. Popko B. Maeda N. Klein A. (1996). Targeted disruption of the mouse sphingolipid activator protein gene: a complex phenotype, including severe leukodystrophy and wide-spread storage of multiple sphingolipids. *Hum. Mol. Genet.* 5 711–725. 10.1093/hmg/5.6.711 8776585

[B84] Furderer M. L. Hertz E. Lopez G. J. Sidransky E. (2022). Neuropathological features of gaucher disease and gaucher disease with Parkinsonism. *Int. J. Mol. Sci.* 23:5842. 10.3390/ijms23105842 35628652 PMC9147326

[B85] Gable K. Gupta S. D. Han G. Niranjanakumari S. Harmon J. M. Dunn T. M. (2010). A disease-causing mutation in the active site of serine palmitoyltransferase causes catalytic promiscuity. *J. Biol. Chem.* 285 22846–22852. 10.1074/jbc.M110.122259 20504773 PMC2906276

[B86] Gantner M. L. Eade K. Wallace M. Handzlik M. K. Fallon R. Trombley J. (2019). Serine and lipid metabolism in macular disease and peripheral neuropathy. *N. Engl. J. Med.* 381 1422–1433. 10.1056/NEJMoa1815111 31509666 PMC7685488

[B87] Garofalo K. Penno A. Schmidt B. P. Lee H. J. Frosch M. P. von Eckardstein A. (2011). Oral L-serine supplementation reduces production of neurotoxic deoxysphingolipids in mice and humans with hereditary sensory autonomic neuropathy type 1. *J. Clin. Invest.* 121 4735–4745. 10.1172/JCI57549 22045570 PMC3225995

[B88] Ghosh A. Kling T. Snaidero N. Sampaio J. L. Shevchenko A. Gras H. (2013). A global in vivo *Drosophila* RNAi screen identifies a key role of ceramide phosphoethanolamine for glial ensheathment of axons. *PLoS Genet.* 9:e1003980. 10.1371/journal.pgen.1003980 24348263 PMC3861124

[B89] Gibbons M. Sarlak S. Chittka L. (2022). Descending control of nociception in insects? *Proc. Biol. Sci.* 289:20220599. 10.1098/rspb.2022.0599 35858073 PMC9257290

[B90] Giguere N. Burke Nanni S. Trudeau L. E. (2018). On cell loss and selective vulnerability of neuronal populations in Parkinson’s disease. *Front. Neurol.* 9:455. 10.3389/fneur.2018.00455 29971039 PMC6018545

[B91] Goutman S. A. Boss J. Guo K. Alakwaa F. M. Patterson A. Kim S. (2020). Untargeted metabolomics yields insight into ALS disease mechanisms. *J. Neurol. Neurosurg. Psychiatry* 91 1329–1338. 10.1136/jnnp-2020-323611 32928939 PMC7677469

[B92] Goutman S. A. Hardiman O. Al-Chalabi A. Chio A. Savelieff M. G. Kiernan M. C. (2022). Emerging insights into the complex genetics and pathophysiology of amyotrophic lateral sclerosis. *Lancet Neurol.* 21 465–479. 10.1016/S1474-4422(21)00414-2 35334234 PMC9513754

[B93] Grabowski G. A. Antommaria A. H. M. Kolodny E. H. Mistry P. K. (2021). Gaucher disease: basic and translational science needs for more complete therapy and management. *Mol. Genet. Metab.* 132 59–75. 10.1016/j.ymgme.2020.12.291 33419694 PMC8809485

[B94] Greffard S. Verny M. Bonnet A. M. Beinis J. Y. Gallinari C. Meaume S. (2006). Motor score of the Unified Parkinson disease rating scale as a good predictor of Lewy body-associated neuronal loss in the substantia nigra. *Arch. Neurol.* 63 584–588. 10.1001/archneur.63.4.584 16606773

[B95] Guadagnolo D. Piane M. Torrisi M. R. Pizzuti A. Petrucci S. (2021). Genotype-phenotype correlations in monogenic Parkinson disease: a review on clinical and molecular findings. *Front. Neurol.* 12:648588. 10.3389/fneur.2021.648588 34630269 PMC8494251

[B96] Guan X. L. Cestra G. Shui G. Kuhrs A. Schittenhelm R. B. Hafen E. (2013). Biochemical membrane lipidomics during *Drosophila* development. *Dev. Cell* 24 98–111. 10.1016/j.devcel.2012.11.012 23260625

[B97] Guedes L. C. Chan R. B. Gomes M. A. Conceicao V. A. Machado R. B. Soares T. (2017). Serum lipid alterations in GBA-associated Parkinson’s disease. *Parkinsonism Relat. Disord.* 44 58–65. 10.1016/j.parkreldis.2017.08.026 28890071

[B98] Guelly C. Zhu P. P. Leonardis L. Papic L. Zidar J. Schabhuttl M. (2011). Targeted high-throughput sequencing identifies mutations in atlastin-1 as a cause of hereditary sensory neuropathy type I. *Am. J. Hum. Genet.* 88 99–105. 10.1016/j.ajhg.2010.12.003 21194679 PMC3014370

[B99] Gupta S. D. Gable K. Alexaki A. Chandris P. Proia R. L. Dunn T. M. (2015). Expression of the ORMDLS, modulators of serine palmitoyltransferase, is regulated by sphingolipids in mammalian cells. *J. Biol. Chem.* 290 90–98. 10.1074/jbc.M114.588236 25395622 PMC4281770

[B100] Haelterman N. A. Yoon W. H. Sandoval H. Jaiswal M. Shulman J. M. Bellen H. J. (2014). A mitocentric view of Parkinson’s disease. *Annu. Rev. Neurosci.* 37 137–159. 10.1146/annurev-neuro-071013-014317 24821430 PMC4659514

[B101] Hammad S. M. Pierce J. S. Soodavar F. Smith K. J. Al Gadban M. M. Rembiesa B. (2010). Blood sphingolipidomics in healthy humans: impact of sample collection methodology. *J. Lipid Res.* 51 3074–3087. 10.1194/jlr.D008532 20660127 PMC2936747

[B102] Han C. Xiong N. Guo X. Huang J. Ma K. Liu L. (2019). Exosomes from patients with Parkinson’s disease are pathological in mice. *J. Mol. Med.* 97 1329–1344. 10.1007/s00109-019-01810-z 31302715

[B103] Hanada K. Kumagai K. Yasuda S. Miura Y. Kawano M. Fukasawa M. (2003). Molecular machinery for non-vesicular trafficking of ceramide. *Nature* 426 803–809. 10.1038/nature02188 14685229

[B104] Harzer K. Paton B. C. Poulos A. Kustermann-Kuhn B. Roggendorf W. Grisar T. (1989). Sphingolipid activator protein deficiency in a 16-week-old atypical Gaucher disease patient and his fetal sibling: biochemical signs of combined sphingolipidoses. *Eur. J. Pediatr.* 149 31–39. 10.1007/BF02024331 2514102

[B105] Healy D. G. Falchi M. O’Sullivan S. S. Bonifati V. Durr A. Bressman S. (2008). Phenotype, genotype, and worldwide genetic penetrance of LRRK2-associated Parkinson’s disease: a case-control study. *Lancet Neurol.* 7 583–590. 10.1016/S1474-4422(08)70117-0 18539534 PMC2832754

[B106] Herr D. R. Fyrst H. Creason M. B. Phan V. H. Saba J. D. Harris G. L. (2004). Characterization of the *Drosophila* sphingosine kinases and requirement for Sk2 in normal reproductive function. *J. Biol. Chem.* 279 12685–12694. 10.1074/jbc.M310647200 14722126

[B107] Herr D. R. Fyrst H. Phan V. Heinecke K. Georges R. Harris G. L. (2003). Sply regulation of sphingolipid signaling molecules is essential for *Drosophila* development. *Development* 130 2443–2453. 10.1242/dev.00456 12702658

[B108] Hewitt V. L. Whitworth A. J. (2017). Mechanisms of Parkinson’s Disease: lessons from *Drosophila*. *Curr. Top. Dev. Biol.* 121 173–200. 10.1016/bs.ctdb.2016.07.005 28057299

[B109] Hindle S. J. Hebbar S. Schwudke D. Elliott C. J. H. Sweeney S. T. (2017). A saposin deficiency model in *Drosophila*: lysosomal storage, progressive neurodegeneration and sensory physiological decline. *Neurobiol. Dis.* 98 77–87. 10.1016/j.nbd.2016.11.012 27913291 PMC5319729

[B110] Hines T. J. Tadenev A. L. D. Lone M. A. Hatton C. L. Bagasrawala I. Stum M. G. (2022). Precision mouse models of Yars/dominant intermediate charcot-marie-tooth disease type C and Sptlc1/hereditary sensory and autonomic neuropathy type 1. *J. Anat.* 241 1169–1185. 10.1111/joa.13605 34875719 PMC9170831

[B111] Hjelmqvist L. Tuson M. Marfany G. Herrero E. Balcells S. Gonzalez-Duarte R. (2002). ORMDL proteins are a conserved new family of endoplasmic reticulum membrane proteins. *Genome Biol.* 3:RESEARCH0027. 10.1186/gb-2002-3-6-research0027 12093374 PMC116724

[B112] Hu Y. Flockhart I. Vinayagam A. Bergwitz C. Berger B. Perrimon N. (2011). An integrative approach to ortholog prediction for disease-focused and other functional studies. *BMC Bioinformatics* 12:357. 10.1186/1471-2105-12-357 21880147 PMC3179972

[B113] Huang X. Warren J. T. Buchanan J. Gilbert L. I. Scott M. P. (2007). *Drosophila* Niemann-Pick type C-2 genes control sterol homeostasis and steroid biosynthesis: a model of human neurodegenerative disease. *Development* 134 3733–3742. 10.1242/dev.004572 17804599

[B114] Hulkova H. Cervenkova M. Ledvinova J. Tochackova M. Hrebicek M. Poupetova H. (2001). A novel mutation in the coding region of the prosaposin gene leads to a complete deficiency of prosaposin and saposins, and is associated with a complex sphingolipidosis dominated by lactosylceramide accumulation. *Hum. Mol. Genet.* 10 927–940. 10.1093/hmg/10.9.927 11309366

[B115] Huttner W. B. Zimmerberg J. (2001). Implications of lipid microdomains for membrane curvature, budding and fission. *Curr. Opin. Cell Biol.* 13 478–484. 10.1016/s0955-0674(00)00239-8 11454455

[B116] Im S. H. Galko M. J. (2012). Pokes, sunburn, and hot sauce: *Drosophila* as an emerging model for the biology of nociception. *Dev. Dyn.* 241 16–26. 10.1002/dvdy.22737 21932321 PMC3258975

[B117] Indelicato E. Nachbauer W. Eigentler A. Amprosi M. Matteucci Gothe R. Giunti P. (2020). Onset features and time to diagnosis in Friedreich’s Ataxia. *Orphanet. J. Rare Dis.* 15:198. 10.1186/s13023-020-01475-9 32746884 PMC7397644

[B118] Inoshita T. Arano T. Hosaka Y. Meng H. Umezaki Y. Kosugi S. (2017). Vps35 in cooperation with LRRK2 regulates synaptic vesicle endocytosis through the endosomal pathway in *Drosophila*. *Hum. Mol. Genet.* 26 2933–2948. 10.1093/hmg/ddx179 28482024

[B119] Iqbal J. Walsh M. T. Hammad S. M. Hussain M. M. (2017). Sphingolipids and lipoproteins in health and metabolic disorders. *Trends Endocrinol. Metab.* 28 506–518. 10.1016/j.tem.2017.03.005 28462811 PMC5474131

[B120] Ishizu N. Yui D. Hebisawa A. Aizawa H. Cui W. Fujita Y. (2016). Impaired striatal dopamine release in homozygous Vps35 D620N knock-in mice. *Hum. Mol. Genet.* 25 4507–4517. 10.1093/hmg/ddw279 28173004

[B121] Iwaki H. Blauwendraat C. Makarious M. B. Bandres-Ciga S. Leonard H. L. Gibbs J. R. (2020). Penetrance of Parkinson’s disease in LRRK2 p.G2019S carriers is modified by a polygenic risk score. *Mov. Disord.* 35 774–780. 10.1002/mds.27974 31958187 PMC8975556

[B122] Johnson J. O. Chia R. Miller D. E. Li R. Kumaran R. Abramzon Y. (2021). Association of variants in the SPTLC1 gene with juvenile amyotrophic lateral sclerosis. *JAMA Neurol.* 78 1236–1248. 10.1001/jamaneurol.2021.2598 34459874 PMC8406220

[B123] Joly J. S. Recher G. Brombin A. Ngo K. Hartenstein V. (2016). A conserved developmental mechanism builds complex visual systems in insects and vertebrates. *Curr. Biol.* 26, R1001–R1009. 10.1016/j.cub.2016.08.017 27780043 PMC5235324

[B124] Kahsai L. Zars T. (2011). Learning and memory in *Drosophila*: Behavior, genetics, and neural systems. *Int. Rev. Neurobiol.* 99 139–167. 10.1016/B978-0-12-387003-2.00006-9 21906539

[B125] Kanca O. Zirin J. Garcia-Marques J. Knight S. M. Yang-Zhou D. Amador G. (2019). An efficient CRISPR-based strategy to insert small and large fragments of DNA using short homology arms. *eLife* 8:e51539. 10.7554/eLife.51539 31674908 PMC6855806

[B126] Kanca O. Zirin J. Hu Y. Tepe B. Dutta D. Lin W. W. (2022). An expanded toolkit for *Drosophila* gene tagging using synthesized homology donor constructs for CRISPR-mediated homologous recombination. *eLife* 11:e76077. 10.7554/eLife.76077 35723254 PMC9239680

[B127] Kelekci S. Yildiz A. B. Sevinc K. Cimen D. U. Onder T. (2022). Perspectives on current models of Friedreich’s ataxia. *Front. Cell Dev. Biol.* 10:958398. 10.3389/fcell.2022.958398 36036008 PMC9403045

[B128] Kim Y. Y. Um J. H. Yoon J. H. Kim H. Lee D. Y. Lee Y. J. (2019). Assessment of mitophagy in mt-Keima *Drosophila* revealed an essential role of the PINK1-Parkin pathway in mitophagy induction in vivo. *FASEB J.* 33 9742–9751. 10.1096/fj.201900073R 31120803 PMC12039051

[B129] Kinghorn K. J. Gronke S. Castillo-Quan J. I. Woodling N. S. Li L. Sirka E. (2016). A *Drosophila* model of neuronopathic gaucher disease demonstrates lysosomal-autophagic defects and altered mTOR signalling and is functionally rescued by rapamycin. *J. Neurosci.* 36 11654–11670. 10.1523/JNEUROSCI.4527-15.2016 27852774 PMC5125225

[B130] Klein C. J. Botuyan M. V. Wu Y. Ward C. J. Nicholson G. A. Hammans S. (2011). Mutations in DNMT1 cause hereditary sensory neuropathy with dementia and hearing loss. *Nat. Genet.* 43 595–600. 10.1038/ng.830 21532572 PMC3102765

[B131] Kohyama-Koganeya A. Sasamura T. Oshima E. Suzuki E. Nishihara S. Ueda R. (2004). *Drosophila* glucosylceramide synthase: a negative regulator of cell death mediated by proapoptotic factors. *J. Biol. Chem.* 279 35995–36002. 10.1074/jbc.M400444200 15210713

[B132] Kornak U. Mademan I. Schinke M. Voigt M. Krawitz P. Hecht J. (2014). Sensory neuropathy with bone destruction due to a mutation in the membrane-shaping atlastin GTPase 3. *Brain* 137(Pt 3), 683–692. 10.1093/brain/awt357 24459106

[B133] Korolchuk V. I. Schutz M. M. Gomez-Llorente C. Rocha J. Lansu N. R. Collins S. M. (2007). *Drosophila* Vps35 function is necessary for normal endocytic trafficking and actin cytoskeleton organisation. *J. Cell Sci.* 120(Pt 24), 4367–4376. 10.1242/jcs.012336 18057029

[B134] Kuchar L. Ledvinova J. Hrebicek M. Myskova H. Dvorakova L. Berna L. (2009). Prosaposin deficiency and saposin B deficiency (activator-deficient metachromatic leukodystrophy): report on two patients detected by analysis of urinary sphingolipids and carrying novel PSAP gene mutations. *Am. J. Med. Genet. A* 149A 613–621. 10.1002/ajmg.a.32712 19267410 PMC3437469

[B135] Lamarche J. B. Cote M. Lemieux B. (1980). The cardiomyopathy of friedreich’s ataxia morphological observations in 3 cases. *Can. J. Neurol. Sci.* 7 389–396. 10.1017/s0317167100022927 6452194

[B136] Lansbury P. (2022). The sphingolipids clearly play a role in Parkinson’s disease, but nature has made it complicated. *Mov. Disord.* 37 1985–1989. 10.1002/mds.29204 36087026

[B137] Lee P. T. Zirin J. Kanca O. Lin W. W. Schulze K. L. Li-Kroeger D. (2018). A gene-specific T2A-GAL4 library for *Drosophila*. *eLife* 7:e35574. 10.7554/eLife.35574 29565247 PMC5898912

[B138] Li H. Janssens J. De Waegeneer M. Kolluru S. S. Davie K. Gardeux V. (2022). Fly Cell Atlas: A single-nucleus transcriptomic atlas of the adult fruit fly. *Science* 375:eabk2432. 10.1126/science.abk2432 35239393 PMC8944923

[B139] Li S. Xie T. Liu P. Wang L. Gong X. (2021). Structural insights into the assembly and substrate selectivity of human SPT-ORMDL3 complex. *Nat. Struct. Mol. Biol.* 28 249–257. 10.1038/s41594-020-00553-7 33558762

[B140] Li-Kroeger D. Kanca O. Lee P. T. Cowan S. Lee M. T. Jaiswal M. (2018). An expanded toolkit for gene tagging based on MiMIC and scarless CRISPR tagging in *Drosophila*. *eLife* 7:e38709. 10.7554/eLife.38709 30091705 PMC6095692

[B141] Lin G. Lee P. T. Chen K. Mao D. Tan K. L. Zuo Z. (2018). Phospholipase PLA2G6, a parkinsonism-associated gene, affects Vps26 and Vps35, retromer function, and ceramide levels, similar to alpha-synuclein gain. *Cell Metab.* 28 605.e6–618.e6. 10.1016/j.cmet.2018.05.019 29909971

[B142] Lin G. Tepe B. McGrane G. Tipon R. C. Croft G. Panwala L. (2023). Exploring therapeutic strategies for Infantile Neuronal Axonal Dystrophy (INAD/PARK14). *bioRxiv* [preprint]. 10.1101/2022.08.16.504080 PMC9889087 36645408

[B143] Lin Z. H. Ruan Y. Xue N. J. Fang Y. Pu J. L. Zhang B. R. (2021). PSAP intronic variants around saposin D domain and Parkinson’s disease. *Brain* 144:e3. 10.1093/brain/awaa354 33197249

[B144] Liu L. MacKenzie K. R. Putluri N. Maletic-Savatic M. Bellen H. J. (2017). The glia-neuron lactate shuttle and elevated ROS promote lipid synthesis in neurons and lipid droplet accumulation in glia via APOE/D. *Cell Metab.* 26 719–737.e6. 10.1016/j.cmet.2017.08.024 28965825 PMC5677551

[B145] Liu L. Zhang K. Sandoval H. Yamamoto S. Jaiswal M. Sanz E. (2015). Glial lipid droplets and ROS induced by mitochondrial defects promote neurodegeneration. *Cell* 160 177–190. 10.1016/j.cell.2014.12.019 25594180 PMC4377295

[B146] Longinetti E. Fang F. (2019). Epidemiology of amyotrophic lateral sclerosis: an update of recent literature. *Curr. Opin. Neurol.* 32 771–776. 10.1097/WCO.0000000000000730 31361627 PMC6735526

[B147] Lu S. Ma M. Mao X. Bacino C. A. Jankovic J. Sutton V. R. (2022). De novo variants in FRMD5 are associated with developmental delay, intellectual disability, ataxia, and abnormalities of eye movement. *Am. J. Hum. Genet.* 109 1932–1943. 10.1016/j.ajhg.2022.09.005 36206744 PMC9606480

[B148] Lu T.-C. Brbiæ M. Park Y.-J. Jackson T. Chen J. Kolluru S. S. (2022). Aging fly cell atlas identifies exhaustive aging features at cellular resolution. *bioRxiv* [Preprint]. 10.1101/2022.12.06.519355 PMC10829769 37319212

[B149] Lubbe S. J. Escott-Price V. Gibbs J. R. Nalls M. A. Bras J. Price T. R. (2016). Additional rare variant analysis in Parkinson’s disease cases with and without known pathogenic mutations: evidence for oligogenic inheritance. *Hum. Mol. Genet.* 25 5483–5489. 10.1093/hmg/ddw348 27798102 PMC5418836

[B150] Ma M. Moulton M. J. Lu S. Bellen H. J. (2022). ‘Fly-ing’ from rare to common neurodegenerative disease mechanisms. *Trends Genet.* 38 972–984. 10.1016/j.tig.2022.03.018 35484057 PMC9378361

[B151] Maio N. Rouault T. A. (2020). Outlining the complex pathway of mammalian Fe-S cluster biogenesis. *Trends Biochem. Sci.* 45 411–426. 10.1016/j.tibs.2020.02.001 32311335 PMC8349188

[B152] Malik B. R. Godena V. K. Whitworth A. J. (2015). VPS35 pathogenic mutations confer no dominant toxicity but partial loss of function in *Drosophila* and genetically interact with parkin. *Hum. Mol. Genet.* 24 6106–6117. 10.1093/hmg/ddv322 26251041 PMC4599670

[B153] Mandala S. M. (2001). Sphingosine-1-phosphate phosphatases. *Prostaglandins Other. Lipid Mediat.* 64 143–156. 10.1016/s0090-6980(01)00111-3 11324704

[B154] Maor G. Cabasso O. Krivoruk O. Rodriguez J. Steller H. Segal D. (2016). The contribution of mutant GBA to the development of Parkinson disease in *Drosophila*. *Hum. Mol. Genet.* 25 2712–2727. 10.1093/hmg/ddw129 27162249 PMC6390410

[B155] Mariano V. Achsel T. Bagni C. Kanellopoulos A. K. (2020). Modelling learning and memory in *Drosophila* to understand intellectual disabilities. *Neuroscience* 445 12–30. 10.1016/j.neuroscience.2020.07.034 32730949

[B156] Masciullo M. Santoro M. Modoni A. Ricci E. Guitton J. Tonali P. (2010). Substrate reduction therapy with miglustat in chronic GM2 gangliosidosis type Sandhoff: results of a 3-year follow-up. *J. Inherit. Metab. Dis.* 33 (Suppl. 3), S355–S361. 10.1007/s10545-010-9186-3 20821051

[B157] Mazzulli J. R. Xu Y. H. Sun Y. Knight A. L. McLean P. J. Caldwell G. A. (2011). Gaucher disease glucocerebrosidase and alpha-synuclein form a bidirectional pathogenic loop in synucleinopathies. *Cell* 146 37–52. 10.1016/j.cell.2011.06.001 21700325 PMC3132082

[B158] McCampbell A. Truong D. Broom D. C. Allchorne A. Gable K. Cutler R. G. (2005). Mutant SPTLC1 dominantly inhibits serine palmitoyltransferase activity in vivo and confers an age-dependent neuropathy. *Hum. Mol. Genet.* 14 3507–3521. 10.1093/hmg/ddi380 16210380

[B159] McWilliams T. G. Muqit M. M. (2017). PINK1 and parkin: emerging themes in mitochondrial homeostasis. *Curr. Opin. Cell Biol.* 45 83–91. 10.1016/j.ceb.2017.03.013 28437683

[B160] Michael S. Petrocine S. V. Qian J. Lamarche J. B. Knutson M. D. Garrick M. D. (2006). Iron and iron-responsive proteins in the cardiomyopathy of Friedreich’s ataxia. *Cerebellum* 5 257–267. 10.1080/14734220600913246 17134988

[B161] Milenkovic I. Blumenreich S. Futerman A. H. (2022). GBA mutations, glucosylceramide and Parkinson’s disease. *Curr. Opin. Neurobiol.* 72 148–154. 10.1016/j.conb.2021.11.004 34883387

[B162] Mistry P. K. Lopez G. Schiffmann R. Barton N. W. Weinreb N. J. Sidransky E. (2017). Gaucher disease: progress and ongoing challenges. *Mol. Genet. Metab.* 120 8–21. 10.1016/j.ymgme.2016.11.006 27916601 PMC5425955

[B163] Miura E. Hasegawa T. Konno M. Suzuki M. Sugeno N. Fujikake N. (2014). VPS35 dysfunction impairs lysosomal degradation of alpha-synuclein and exacerbates neurotoxicity in a *Drosophila* model of Parkinson’s disease. *Neurobiol. Dis.* 71 1–13. 10.1016/j.nbd.2014.07.014 25107340

[B164] Modi M. N. Shuai Y. Turner G. C. (2020). The *Drosophila* mushroom body: From architecture to algorithm in a learning circuit. *Annu. Rev. Neurosci.* 43 465–484. 10.1146/annurev-neuro-080317-0621333 32283995

[B165] Mohassel P. Donkervoort S. Lone M. A. Nalls M. Gable K. Gupta S. D. (2021). Childhood amyotrophic lateral sclerosis caused by excess sphingolipid synthesis. *Nat. Med.* 27 1197–1204. 10.1038/s41591-021-01346-1 34059824 PMC9309980

[B166] Mollinari C. Zhao J. Lupacchini L. Garaci E. Merlo D. Pei G. (2018). Transdifferentiation: a new promise for neurodegenerative diseases. *Cell Death Dis.* 9:830. 10.1038/s41419-018-0891-4 30082779 PMC6078988

[B167] Monnier V. Llorens J. V. Navarro J. A. (2018). Impact of *Drosophila* models in the study and treatment of Friedreich’s ataxia. *Int. J. Mol. Sci.* 19:1989. 10.3390/ijms19071989 29986523 PMC6073496

[B168] Monzio Compagnoni G. Di Fonzo A. Corti S. Comi G. P. Bresolin N. Masliah E. (2020). The role of mitochondria in Neurodegenerative diseases: the lesson from Alzheimer’s disease and Parkinson’s disease. *Mol. Neurobiol.* 57 2959–2980. 10.1007/s12035-020-01926-1 32445085 PMC9047992

[B169] Moser J. M. Bigini P. Schmitt-John T. (2013). The wobbler mouse, an ALS animal model. *Mol. Genet. Genomics* 288 207–229. 10.1007/s00438-013-0741-0 23539154 PMC3664746

[B170] Moulton M. J. Barish S. Ralhan I. Chang J. Goodman L. D. Harland J. G. (2021). Neuronal ROS-induced glial lipid droplet formation is altered by loss of Alzheimer’s disease-associated genes. *Proc. Natl. Acad. Sci. U.S.A.* 118:e2112095118. 10.1073/pnas.2112095118 34949639 PMC8719885

[B171] Nagarkar-Jaiswal S. DeLuca S. Z. Lee P. T. Lin W. W. Pan H. Zuo Z. (2015). A genetic toolkit for tagging intronic MiMIC containing genes. *eLife* 4:e08469. 10.7554/eLife.08469 26102525 PMC4499919

[B172] Nalls M. A. Blauwendraat C. Vallerga C. L. Heilbron K. Bandres-Ciga S. Chang D. (2019). Identification of novel risk loci, causal insights, and heritable risk for Parkinson’s disease: a meta-analysis of genome-wide association studies. *Lancet Neurol.* 18 1091–1102. 10.1016/S1474-4422(19)30320-5 31701892 PMC8422160

[B173] Navarro J. A. Ohmann E. Sanchez D. Botella J. A. Liebisch G. Molto M. D. (2010). Altered lipid metabolism in a *Drosophila* model of Friedreich’s ataxia. *Hum. Mol. Genet.* 19 2828–2840. 10.1093/hmg/ddq183 20460268 PMC7108586

[B174] Newton J. Milstien S. Spiegel S. (2018). Niemann-Pick type C disease: the atypical sphingolipidosis. *Adv. Biol. Regul.* 70 82–88. 10.1016/j.jbior.2018.08.001 30205942 PMC6327306

[B175] Nixon R. A. (2020). The aging lysosome: an essential catalyst for late-onset neurodegenerative diseases. *Biochim. Biophys. Acta Proteins Proteom.* 1868:140443. 10.1016/j.bbapap.2020.140443 32416272 PMC7388076

[B176] Noval S. Contreras I. Sanz-Gallego I. Manrique R. K. Arpa J. (2012). Ophthalmic features of Friedreich ataxia. *Eye* 26 315–320. 10.1038/eye.2011.291 22094302 PMC3272198

[B177] O’Brien J. S. Kishimoto Y. (1991). Saposin proteins: structure, function, and role in human lysosomal storage disorders. *FASEB J.* 5 301–308. 10.1096/fasebj.5.3.2001789 2001789

[B178] Oji Y. Hatano T. Ueno S. I. Funayama M. Ishikawa K. I. Okuzumi A. (2020b). Variants in saposin D domain of prosaposin gene linked to Parkinson’s disease. *Brain* 143 1190–1205. 10.1093/brain/awaa064 32201884

[B179] Oji Y. Hatano T. Funayama M. Hattori N. (2020a). Reply: saposin D variants are not a common cause of familial Parkinson’s disease among Italians; and Lack of evidence for genetic association of saposins A. B, C and D with Parkinson’s disease. *Brain* 143:e73. 10.1093/brain/awaa215 32793945

[B180] Oji Y. Hatano T. Funayama M. Hattori N. (2021a). Reply: association analysis of PSAP variants in Parkinson’s disease patients. *Brain* 144:e10. 10.1093/brain/awaa359 33221836

[B181] Oji Y. Hatano T. Funayama M. Hattori N. (2021b). Reply: PSAP intronic variants around saposin D domain and Parkinson’s disease. *Brain* 144:e4. 10.1093/brain/awaa356 33197238

[B182] Oji Y. Hatano T. Funayama M. Hattori N. (2021c). Reply: PSAP variants in Parkinson’s disease: a large cohort study in Chinese mainland population. *Brain* 144:e26. 10.1093/brain/awaa393 33844829

[B183] Osawa Y. Banno Y. Nagaki M. Brenner D. A. Naiki T. Nozawa Y. (2001). TNF-alpha-induced sphingosine 1-phosphate inhibits apoptosis through a phosphatidylinositol 3-kinase/Akt pathway in human hepatocytes. *J. Immunol.* 167 173–180. 10.4049/jimmunol.167.1.173 11418646

[B184] Osuchowski M. F. Johnson V. J. He Q. Sharma R. P. (2004). Myriocin, a serine palmitoyltransferase inhibitor, alters regional brain neurotransmitter levels without concurrent inhibition of the brain sphingolipid biosynthesis in mice. *Toxicol. Lett.* 147 87–94. 10.1016/j.toxlet.2003.10.016 14700532

[B185] Oswald M. C. West R. J. Lloyd-Evans E. Sweeney S. T. (2015). Identification of dietary alanine toxicity and trafficking dysfunction in a *Drosophila* model of hereditary sensory and autonomic neuropathy type 1. *Hum. Mol. Genet.* 24 6899–6909. 10.1093/hmg/ddv390 26395456 PMC4654049

[B186] Oya Y. Nakayasu H. Fujita N. Suzuki K. Suzuki K. (1998). Pathological study of mice with total deficiency of sphingolipid activator proteins (SAP knockout mice). *Acta Neuropathol.* 96 29–40. 10.1007/s004010050857 9678511

[B187] Panza E. Meyyazhagan A. Orlacchio A. (2022). Hereditary spastic paraplegia: genetic heterogeneity and common pathways. *Exp. Neurol.* 357:114203. 10.1016/j.expneurol.2022.114203 35970204

[B188] Papadopoulos V. E. Nikolopoulou G. Antoniadou I. Karachaliou A. Arianoglou G. Emmanouilidou E. (2018). Modulation of beta-glucocerebrosidase increases alpha-synuclein secretion and exosome release in mouse models of Parkinson’s disease. *Hum. Mol. Genet.* 27 1696–1710. 10.1093/hmg/ddy075 29547959

[B189] Park J. Lee S. B. Lee S. Kim Y. Song S. Kim S. (2006). Mitochondrial dysfunction in *Drosophila* PINK1 mutants is complemented by parkin. *Nature* 441 1157–1161. 10.1038/nature04788 16672980

[B190] Patterson M. C. Vecchio D. Prady H. Abel L. Wraith J. E. (2007). Miglustat for treatment of Niemann-Pick C disease: a randomised controlled study. *Lancet Neurol.* 6 765–772. 10.1016/S1474-4422(07)70194-1 17689147

[B191] Paul S. Pickrell A. M. (2021). Hidden phenotypes of PINK1/Parkin knockout mice. *Biochim. Biophys. Acta Gen. Subj.* 1865:129871. 10.1016/j.bbagen.2021.129871 33571581

[B192] Penno A. Reilly M. M. Houlden H. Laura M. Rentsch K. Niederkofler V. (2010). Hereditary sensory neuropathy type 1 is caused by the accumulation of two neurotoxic sphingolipids. *J. Biol. Chem.* 285 11178–11187. 10.1074/jbc.M109.092973 20097765 PMC2856995

[B193] Perry M. Konstantinides N. Pinto-Teixeira F. Desplan C. (2017). Generation and evolution of neural cell types and circuits: Insights from the *Drosophila* visual system. *Annu. Rev. Genet.* 51 501–527. 10.1146/annurev-genet-120215-035312 28961025 PMC5849253

[B194] Petit C. S. Lee J. J. Boland S. Swarup S. Christiano R. Lai Z. W. (2020). Inhibition of sphingolipid synthesis improves outcomes and survival in GARP mutant wobbler mice, a model of motor neuron degeneration. *Proc. Natl. Acad. Sci. U.S.A.* 117 10565–10574. 10.1073/pnas.1913956117 32345721 PMC7229683

[B195] Pfeiffenberger C. Lear B. C. Keegan K. P. Allada R. (2010). Locomotor activity level monitoring using the *Drosophila* activity monitoring (DAM) system. *Cold Spring Harb. Protoc.* 2010:dbrot5518. 10.1101/pdb.prot5518 21041391

[B196] Phelps J. S. Hildebrand D. G. C. Graham B. J. Kuan A. T. Thomas L. A. Nguyen T. M. (2021). Reconstruction of motor control circuits in adult *Drosophila* using automated transmission electron microscopy. *Cell* 184 759–774.e18. 10.1016/j.cell.2020.12.013 33400916 PMC8312698

[B197] Phillips S. E. Woodruff E. A. III Liang P. Patten M. Broadie K. (2008). Neuronal loss of *Drosophila* NPC1a causes cholesterol aggregation and age-progressive neurodegeneration. *J. Neurosci.* 28 6569–6582. 10.1523/JNEUROSCI.5529-07.2008 18579730 PMC3306184

[B198] Pihlstrom L. Toft M. (2011). Genetic variability in SNCA and Parkinson’s disease. *Neurogenetics* 12 283–293. 10.1007/s10048-011-0292-7 21800132

[B199] Platt F. M. d’Azzo A. Davidson B. L. Neufeld E. F. Tifft C. J. (2018). Lysosomal storage diseases. *Nat. Rev. Dis. Primers* 4:27. 10.1038/s41572-018-0025-4 30275469

[B200] Polymeropoulos M. H. Lavedan C. Leroy E. Ide S. E. Dehejia A. Dutra A. (1997). Mutation in the alpha-synuclein gene identified in families with Parkinson’s disease. *Science* 276 2045–2047. 10.1126/science.276.5321.2045 9197268

[B201] Pool A. H. Scott K. (2014). Feeding regulation in *Drosophila*. *Curr. Opin. Neurobiol.* 29 57–63. 10.1016/j.conb.2014.05.008 24937262 PMC4253568

[B202] Poole A. C. Thomas R. E. Andrews L. A. McBride H. M. Whitworth A. J. Pallanck L. J. (2008). The PINK1/Parkin pathway regulates mitochondrial morphology. *Proc. Natl. Acad. Sci. U.S.A.* 105 1638–1643. 10.1073/pnas.0709336105 18230723 PMC2234197

[B203] Prasad R. Hadjidemetriou I. Maharaj A. Meimaridou E. Buonocore F. Saleem M. (2017). Sphingosine-1-phosphate lyase mutations cause primary adrenal insufficiency and steroid-resistant nephrotic syndrome. *J. Clin. Invest.* 127 942–953. 10.1172/JCI90171 28165343 PMC5330744

[B204] Pruett S. T. Bushnev A. Hagedorn K. Adiga M. Haynes C. A. Sullards M. C. (2008). Biodiversity of sphingoid bases (”sphingosines”) and related amino alcohols. *J. Lipid Res.* 49 1621–1639. 10.1194/jlr.R800012-JLR200 18499644 PMC2444003

[B205] Ramdya P. Benton R. (2010). Evolving olfactory systems on the fly. *Trends Genet.* 26 307–316. 10.1016/j.tig.2010.04.004 20537755

[B206] Rao R. P. Yuan C. Allegood J. C. Rawat S. S. Edwards M. B. Wang X. (2007). Ceramide transfer protein function is essential for normal oxidative stress response and lifespan. *Proc. Natl. Acad. Sci. U.S.A.* 104 11364–11369. 10.1073/pnas.0705049104 17592126 PMC1899189

[B207] Ren M. Yang Y. Heng K. H. Y. Ng L. Y. Chong C. Y. Ng Y. T. (2022). MED13 and glycolysis are conserved modifiers of alpha-synuclein-associated neurodegeneration. *Cell Rep.* 41:111852. 10.1016/j.celrep.2022.111852 36543134

[B208] Rietveld A. Neutz S. Simons K. Eaton S. (1999). Association of sterol- and glycosylphosphatidylinositol-linked proteins with *Drosophila* raft lipid microdomains. *J. Biol. Chem.* 274 12049–12054. 10.1074/jbc.274.17.12049 10207028

[B209] Robak L. A. Jansen I. E. van Rooij J. Uitterlinden A. G. Kraaij R. Jankovic J. (2017). Excessive burden of lysosomal storage disorder gene variants in Parkinson’s disease. *Brain* 140 3191–3203. 10.1093/brain/awx285 29140481 PMC5841393

[B210] Rocha E. M. Keeney M. T. Di Maio R. De Miranda B. R. Greenamyre J. T. (2022). LRRK2 and idiopathic Parkinson’s disease. *Trends Neurosci.* 45 224–236. 10.1016/j.tins.2021.12.002 34991886 PMC8854345

[B211] Roshan Lal T. Sidransky E. (2017). The spectrum of neurological manifestations associated with gaucher disease. *Diseases* 5:10. 10.3390/diseases5010010 28933363 PMC5456331

[B212] Roszczyc-Owsiejczuk K. Zabielski P. (2021). Sphingolipids as a culprit of mitochondrial dysfunction in insulin resistance and type 2 diabetes. *Front. Endocrinol.* 12:635175. 10.3389/fendo.2021.635175 33815291 PMC8013882

[B213] Rotthier A. Auer-Grumbach M. Janssens K. Baets J. Penno A. Almeida-Souza L. (2010). Mutations in the SPTLC2 subunit of serine palmitoyltransferase cause hereditary sensory and autonomic neuropathy type I. *Am. J. Hum. Genet.* 87 513–522. 10.1016/j.ajhg.2010.09.010 20920666 PMC2948807

[B214] Rousseaux M. W. C. Vazquez-Velez G. E. Al-Ramahi I. Jeong H. H. Bajic A. Revelli J. P. (2018). A druggable genome screen identifies modifiers of alpha-synuclein levels via a tiered cross-species validation approach. *J. Neurosci.* 38 9286–9301. 10.1523/JNEUROSCI.0254-18.2018 30249792 PMC6199406

[B215] Russo S. B. Ross J. S. Cowart L. A. (2013). “Sphingolipids in obesity, type 2 diabetes, and metabolic disease,” in *Sphingolipids in Disease. Handbook of Experimental Pharmacology*, Vol. 216, eds Gulbins E. Petrache I. (Vienna: Springer). 10.1007/978-3-7091-1511-4_19 PMC4091661 23563667

[B216] Schapira A. H. V. Chaudhuri K. R. Jenner P. (2017). Non-motor features of Parkinson disease. *Nat. Rev. Neurosci.* 18 435–450. 10.1038/nrn.2017.62 28592904

[B217] Schiffmann R. Fitzgibbon E. J. Harris C. DeVile C. Davies E. H. Abel L. (2008). Randomized, controlled trial of miglustat in Gaucher’s disease type 3. *Ann. Neurol.* 64 514–522. 10.1002/ana.21491 19067373 PMC2605167

[B218] Schrader M. Fahimi H. D. (2006). Peroxisomes and oxidative stress. *Biochim. Biophy.s Acta* 1763 1755–1766. 10.1016/j.bbamcr.2006.09.006 17034877

[B219] Sellin J. Schulze H. Paradis M. Gosejacob D. Papan C. Shevchenko A. (2017). Characterization of *Drosophila* Saposin-related mutants as a model for lysosomal sphingolipid storage diseases. *Dis. Model. Mech.* 10 737–750. 10.1242/dmm.027953 28389479 PMC5483003

[B220] Sidransky E. Nalls M. A. Aasly J. O. Aharon-Peretz J. Annesi G. Barbosa E. R. (2009). Multicenter analysis of glucocerebrosidase mutations in Parkinson’s disease. *N. Engl. J. Med.* 361 1651–1661. 10.1056/NEJMoa0901281 19846850 PMC2856322

[B221] Singh I. Moser A. E. Goldfischer S. Moser H. W. (1984). Lignoceric acid is oxidized in the peroxisome: implications for the Zellweger cerebro-hepato-renal syndrome and adrenoleukodystrophy. *Proc. Natl. Acad. Sci. U.S.A.* 81 4203–4207. 10.1073/pnas.81.13.4203 6588384 PMC345397

[B222] Siow D. L. Wattenberg B. W. (2012). Mammalian ORMDL proteins mediate the feedback response in ceramide biosynthesis. *J. Biol. Chem.* 287 40198–40204. 10.1074/jbc.C112.404012 23066021 PMC3504734

[B223] Small S. A. Petsko G. A. (2015). Retromer in Alzheimer disease, Parkinson disease and other neurological disorders. *Nat. Rev. Neurosci.* 16 126–132. 10.1038/nrn3896 25669742

[B224] Sol J. Jove M. Povedano M. Sproviero W. Dominguez R. Pinol-Ripoll G. (2021). Lipidomic traits of plasma and cerebrospinal fluid in amyotrophic lateral sclerosis correlate with disease progression. *Brain Commun.* 3:fcab143. 10.1093/braincomms/fcab143 34396104 PMC8361390

[B225] Sosero Y. L. Bandres-Ciga S. Hassin-Baer S. Alcalay R. N. Gan-Or Z. International Parkinson’s Disease Genomics Consortium (IPDGC) (2020). Lack of evidence for genetic association of saposins A, B, C and D with Parkinson’s disease. *Brain* 143:e72. 10.1093/brain/awaa214 32793944 PMC7523699

[B226] Soto C. Pritzkow S. (2018). Protein misfolding, aggregation, and conformational strains in neurodegenerative diseases. *Nat. Neurosci.* 21 1332–1340. 10.1038/s41593-018-0235-9 30250260 PMC6432913

[B227] Spiegel S. Milstien S. (2011). The outs and the ins of sphingosine-1-phosphate in immunity. *Nat. Rev. Immunol.* 11 403–415. 10.1038/nri2974 21546914 PMC3368251

[B228] Srivastava S. Mor Shaked H. Gable K. Gupta S. D. Pan X. Somashekarappa N. (2023). SPTSSA variants alter sphingolipid synthesis and cause a complex hereditary spastic paraplegia. *Brain* [preprint]. 10.1093/brain/awac460 PMC10319774 36718090

[B229] Steiner J. A. Angot E. Brundin P. (2011). A deadly spread: cellular mechanisms of alpha-synuclein transfer. *Cell Death Differ.* 18 1425–1433. 10.1038/cdd.2011.53 21566660 PMC3178422

[B230] Stepien K. M. Roncaroli F. Turton N. Hendriksz C. J. Roberts M. Heaton R. A. (2020). Mechanisms of mitochondrial dysfunction in lysosomal storage disorders: a review. *J. Clin. Med.* 9:2596. 10.3390/jcm9082596 32796538 PMC7463786

[B231] Straniero L. Rimoldi V. Monfrini E. Bonvegna S. Melistaccio G. Lake J. (2022). Role of lysosomal gene variants in modulating GBA-associated Parkinson’s disease risk. *Mov. Disord.* 37 1202–1210. 10.1002/mds.28987 35262230 PMC9310717

[B232] Sulzer D. Edwards R. H. (2019). The physiological role of alpha-synuclein and its relationship to Parkinson’s Disease. *J. Neurochem.* 150 475–486. 10.1111/jnc.14810 31269263 PMC6707892

[B233] Tallaksen C. M. Berg J. E. (2009). Miglustat therapy in juvenile Sandhoff disease. *J. Inherit. Metab. Dis.* 32 (Suppl. 1), S289–S293. 10.1007/s10545-009-1224-7 19898953

[B234] Ternes P. Franke S. Zahringer U. Sperling P. Heinz E. (2002). Identification and characterization of a sphingolipid delta 4-desaturase family. *J. Biol. Chem.* 277 25512–25518. 10.1074/jbc.M202947200 11937514

[B235] Thomas R. E. Vincow E. S. Merrihew G. E. MacCoss M. J. Davis M. Y. Pallanck L. J. (2018). Glucocerebrosidase deficiency promotes protein aggregation through dysregulation of extracellular vesicles. *PLoS Genet.* 14:e1007694. 10.1371/journal.pgen.1007694 30256786 PMC6175534

[B236] Trinh K. Moore K. Wes P. D. Muchowski P. J. Dey J. Andrews L. (2008). Induction of the phase II detoxification pathway suppresses neuron loss in *Drosophila* models of Parkinson’s disease. *J. Neurosci.* 28 465–472. 10.1523/JNEUROSCI.4778-07.2008 18184789 PMC6670551

[B237] Tuthill J. C. Wilson R. I. (2016). Mechanosensation and adaptive motor control in insects. *Curr. Biol.* 26 R1022–R1038. 10.1016/j.cub.2016.06.070 27780045 PMC5120761

[B238] Ugur B. Chen K. Bellen H. J. (2016). *Drosophila* tools and assays for the study of human diseases. *Dis. Model. Mech.* 9 235–244. 10.1242/dmm.023762 26935102 PMC4833332

[B239] Vacaru A. M. van den Dikkenberg J. Ternes P. Holthuis J. C. (2013). Ceramide phosphoethanolamine biosynthesis in *Drosophila* is mediated by a unique ethanolamine phosphotransferase in the Golgi lumen. *J. Biol. Chem.* 288 11520–11530. 10.1074/jbc.M113.460972 23449981 PMC3630839

[B240] Vazquez-Velez G. E. Zoghbi H. Y. (2021). Parkinson’s disease genetics and pathophysiology. *Annu. Rev. Neurosci.* 44 87–108. 10.1146/annurev-neuro-100720-034518 34236893

[B241] Verstraeten A. Theuns J. Van Broeckhoven C. (2015). Progress in unraveling the genetic etiology of Parkinson disease in a genomic era. *Trends Genet.* 31 140–149. 10.1016/j.tig.2015.01.004 25703649

[B242] Vilarino-Guell C. Wider C. Ross O. A. Dachsel J. C. Kachergus J. M. Lincoln S. J. (2011). VPS35 mutations in Parkinson disease. *Am. J. Hum. Genet.* 89 162–167. 10.1016/j.ajhg.2011.06.001 21763482 PMC3135796

[B243] Vos M. Dulovic-Mahlow M. Mandik F. Frese L. Kanana Y. Haissatou Diaw S. (2021). Ceramide accumulation induces mitophagy and impairs beta-oxidation in PINK1 deficiency. *Proc. Natl. Acad. Sci. U.S.A.* 118:e2025347118. 10.1073/pnas.2025347118 34686591 PMC8639384

[B244] Waldvogel D. van Gelderen P. Hallett M. (1999). Increased iron in the dentate nucleus of patients with Friedrich’s ataxia. *Ann. Neurol.* 46 123–125. 10.1002/1531-8249(199907)46:1<123::aid-ana19>3.0.co;2-h. 10401790

[B245] Walkley S. U. (2004). Secondary accumulation of gangliosides in lysosomal storage disorders. *Semin. Cell Dev. Biol.* 15 433–444. 10.1016/j.semcdb.2004.03.002 15207833

[B246] Wallings R. L. Humble S. W. Ward M. E. Wade-Martins R. (2019). Lysosomal dysfunction at the centre of Parkinson’s disease and frontotemporal dementia/amyotrophic lateral sclerosis. *Trends Neurosci.* 42 899–912. 10.1016/j.tins.2019.10.002 31704179 PMC6931156

[B247] Wandall H. H. Pedersen J. W. Park C. Levery S. B. Pizette S. Cohen S. M. (2003). *Drosophila* egghead encodes a beta 1,4-mannosyltransferase predicted to form the immediate precursor glycosphingolipid substrate for brainiac. *J. Biol. Chem.* 278 1411–1414. 10.1074/jbc.C200619200 12454022

[B248] Wandall H. H. Pizette S. Pedersen J. W. Eichert H. Levery S. B. Mandel U. (2005). Egghead and brainiac are essential for glycosphingolipid biosynthesis in vivo. *J. Biol. Chem.* 280 4858–4863. 10.1074/jbc.C400571200 15611100

[B249] Wang C. Telpoukhovskaia M. A. Bahr B. A. Chen X. Gan L. (2018). Endo-lysosomal dysfunction: a converging mechanism in neurodegenerative diseases. *Curr. Opin. Neurobiol.* 48 52–58. 10.1016/j.conb.2017.09.005 29028540

[B250] Wang L. Lin G. Zuo Z. Li Y. Byeon S. K. Pandey A. (2022c). Neuronal activity induces glucosylceramide that is secreted via exosomes for lysosomal degradation in glia. *Sci. Adv.* 8:eabn3326. 10.1126/sciadv.abn3326 35857503 PMC9278864

[B251] Wang D. Ho E. S. Cotticelli M. G. Xu P. Napierala J. S. Hauser L. A. (2022b). Skin fibroblast metabolomic profiling reveals that lipid dysfunction predicts the severity of Friedreich’s ataxia. *J. Lipid Res.* 63:100255. 10.1016/j.jlr.2022.100255 35850241 PMC9399481

[B252] Wang D. Cotticelli M. G. Himes B. E. Lynch D. R. Mesaros C. (2022a). Plasma multi-omics analysis reveals very long chain ceramides as validated biomarkers of Friedreich’s ataxia. *medRxiv* [preprint]. 10.1101/2022.09.27.22280432

[B253] Wang D. Qian L. Xiong H. Liu J. Neckameyer W. S. Oldham S. (2006). Antioxidants protect PINK1-dependent dopaminergic neurons in *Drosophila*. *Proc. Natl. Acad. Sci. U.S.A.* 103 13520–13525. 10.1073/pnas.0604661103 16938835 PMC1569195

[B254] Wang S. Tan K. L. Agosto M. A. Xiong B. Yamamoto S. Sandoval H. (2014). The retromer complex is required for rhodopsin recycling and its loss leads to photoreceptor degeneration. *PLoS Biol.* 12:e1001847. 10.1371/journal.pbio.1001847 24781186 PMC4004542

[B255] Wang Y. Niu Y. Zhang Z. Gable K. Gupta S. D. Somashekarappa N. (2021). Structural insights into the regulation of human serine palmitoyltransferase complexes. *Nat. Struct. Mol. Biol.* 28 240–248. 10.1038/s41594-020-00551-9 33558761 PMC9812531

[B256] Wangler M. F. Chao Y. H. Bayat V. Giagtzoglou N. Shinde A. B. Putluri N. (2017). Peroxisomal biogenesis is genetically and biochemically linked to carbohydrate metabolism in *Drosophila* and mouse. *PLoS Genet.* 13:e1006825. 10.1371/journal.pgen.1006825 28640802 PMC5480855

[B257] Ward P. G. D. Harding I. H. Close T. G. Corben L. A. Delatycki M. B. Storey E. (2019). Longitudinal evaluation of iron concentration and atrophy in the dentate nuclei in friedreich ataxia. *Mov. Disord.* 34 335–343. 10.1002/mds.27606 30624809

[B258] Yalcin B. Zhao L. Stofanko M. O’Sullivan N. C. Kang Z. H. Roost A. (2017). Modeling of axonal endoplasmic reticulum network by spastic paraplegia proteins. *Elife* 6:e23882. 10.7554/eLife.23882 28742022 PMC5576921

[B259] Yamamoto S. Jaiswal M. Charng W. L. Gambin T. Karaca E. Mirzaa G. (2014). A *drosophila* genetic resource of mutants to study mechanisms underlying human genetic diseases. *Cell* 159 200–214. 10.1016/j.cell.2014.09.002 25259927 PMC4298142

[B260] Yang Y. Gehrke S. Imai Y. Huang Z. Ouyang Y. Wang J. W. (2006). Mitochondrial pathology and muscle and dopaminergic neuron degeneration caused by inactivation of *Drosophila* Pink1 is rescued by Parkin. *Proc. Natl. Acad. Sci. U.S.A.* 103 10793–10798. 10.1073/pnas.0602493103 16818890 PMC1502310

[B261] Yang Y. Ouyang Y. Yang L. Beal M. F. McQuibban A. Vogel H. (2008). Pink1 regulates mitochondrial dynamics through interaction with the fission/fusion machinery. *Proc. Natl. Acad. Sci. U.S.A.* 105 7070–7075. 10.1073/pnas.0711845105 18443288 PMC2383971

[B262] Yarmolinsky D. A. Zuker C. S. Ryba N. J. (2009). Common sense about taste: From mammals to insects. *Cell* 139 234–244. 10.1016/j.cell.2009.10.001 19837029 PMC3936514

[B263] Ye H. Ojelade S. A. Li-Kroeger D. Zuo Z. Wang L. Li Y. (2020). Retromer subunit, VPS29, regulates synaptic transmission and is required for endolysosomal function in the aging brain. *eLife* 9:e51977. 10.7554/eLife.51977 32286230 PMC7182434

[B264] Ye H. Robak L. A. Yu M. Cykowski M. Shulman J. M. (2022). Genetics and pathogenesis of Parkinson’s syndrome. *Annu. Rev. Pathol.* 18 95–121. 10.1146/annurev-pathmechdis-031521-034145 36100231 PMC10290758

[B265] Yuan C. Rao R. P. Jesmin N. Bamba T. Nagashima K. Pascual A. (2011). CDase is a pan-ceramidase in *Drosophila*. *Mol. Biol. Cell* 22 33–43. 10.1091/mbc.E10-05-0453 21148295 PMC3016975

[B266] Zarin A. A. Mark B. Cardona A. Litwin-Kumar A. Doe C. Q. (2019). A multilayer circuit architecture for the generation of distinct locomotor behaviors in *Drosophila*. *Elife* 8:e51781. 10.7554/eLife.51781 31868582 PMC6994239

[B267] Zhao L. Spassieva S. Gable K. Gupta S. D. Shi L. Y. Wang J. (2015). Elevation of 20-carbon long chain bases due to a mutation in serine palmitoyltransferase small subunit b results in neurodegeneration. *Proc. Natl. Acad. Sci. U.S.A.* 112 12962–12967. 10.1073/pnas.1516733112 26438849 PMC4620873

[B268] Zhao Y. W. Pan H. X. Zeng Q. Fang Z. H. Liu Z. H. Wang Y. (2021). PSAP variants in Parkinson’s disease: a large cohort study in Chinese mainland population. *Brain* 144:e25. 10.1093/brain/awaa391 33793763

